# Black-Winged Kite Algorithm Integrating Opposition-Based Learning and Quasi-Newton Strategy

**DOI:** 10.3390/biomimetics11010068

**Published:** 2026-01-14

**Authors:** Ning Zhao, Tinghua Wang, Yating Zhu

**Affiliations:** Key Laboratory of Data Science and Artificial Intelligence of Jiangxi Education Institutes, Gannan Normal University, Ganzhou 341000, China; 1230741004@gnnu.edu.cn (N.Z.);

**Keywords:** black-winged kite algorithm, optimization, opposition-based learning, quasi-Newton strategy, engineering problem

## Abstract

To address the deficiencies in global search capability and population diversity decline of the black-winged kite algorithm (BKA), this paper proposes an enhanced black-winged kite algorithm integrating opposition-based learning and quasi-Newton strategy (OQBKA). The algorithm introduces a mirror imaging strategy based on convex lens imaging (MOBL) during the migration phase to enhance the population’s spatial distribution and assist individuals in escaping local optima. In later iterations, it incorporates the quasi-Newton method to enhance local optimization precision and convergence performance. Ablation studies on the CEC2017 benchmark set confirm the strong complementarity between the two integrated strategies, with OQBKA achieving an average ranking of 1.34 across all 29 test functions. Comparative experiments on the CEC2022 benchmark suite further verify its superior exploration–exploitation balance and optimization accuracy: under 10- and 20-dimensional settings, OQBKA attains the best average rankings of 2.5 and 2.17 across all 12 test functions, outperforming ten state-of-the-art metaheuristic algorithms. Moreover, evaluations on three constrained engineering design problems, including step-cone pulley optimization, corrugated bulkhead design, and reactor network design, demonstrate the practicality and robustness of the proposed approach in generating feasible solutions under complex constraints.

## 1. Introduction

As an important branch of artificial intelligence, swarm intelligence algorithms [1] have become a crucial tool for solving complex optimization problems due to their strong global search capability and good adaptability. In the face of challenges such as multi-objective optimization and uncertainty, traditional optimization algorithms often fail to meet the requirements. In contrast, swarm intelligence algorithms, with their flexibility and gradient-free mechanism, can achieve global optimization in complex search spaces. They have been widely applied in fields such as engineering problems, machine learning, and data mining [2,3], where they demonstrate remarkable advantages. Common swarm intelligence algorithms include particle swarm optimization (PSO) [4], harris hawks optimizer (HHO) [5], grey wolf optimizer (GWO) [6], and sparrow search algorithm (SSA) [7].

In recent years, inspired by natural phenomena and collective behaviors, numerous emerging swarm intelligence algorithms have been proposed, offering new feasible approaches for addressing complex optimization tasks. Examples include the artificial lemming algorithm (ALA) [8], mirage search optimization (MSO) [9], whale migration algorithm (WMA) [10], and black-winged kite algorithm (BKA) [11]. Among them, the BKA is a swarm intelligence optimization algorithm inspired by the migration and predation behaviors of black-winged kites. By integrating the Cauchy mutation strategy and leader-based mechanism, BKA enhances global search capability while significantly improving convergence speed, thereby achieving a good balance between global exploration and local exploitation. However, BKA suffers from insufficient population diversity, making it prone to premature convergence or repetitive convergence, which limits its performance when dealing with complex optimization problems in high-dimensional search spaces.

To address the aforementioned shortcomings, numerous researchers have proposed improvements to the BKA. Dai et al. [12] proposed the hybrid black-winged kite algorithm (BWOA), which combines the precise search mechanism of BKA with the spiral encircling strategy of the whale optimization algorithm (WOA). By dividing high-fitness individuals into subgroups for parallel optimization and introducing an elitism mechanism and levy flight perturbation, the algorithm effectively balances global exploration and local exploitation, preventing premature convergence. Zhang et al. [13] proposed the osprey-combined black-winged kite algorithm (OCBKA), which uses Logistic chaotic mapping for population initialization and integrates the osprey optimization algorithm (OOA) to enhance global search ability and overall performance. Zhou et al. [14] proposed the BKA optimization algorithm based on sine-cosine guidelines (SCBKA), which significantly improves convergence speed and solution accuracy. Zhang et al. [15] developed an Improved Black-winged Kite Algorithm (IBKA) for network security situation assessment. Their method integrates Gaussian mutation, opposition-based learning, and an optimal individual oscillation strategy, achieving superior convergence accuracy compared with other swarm intelligence algorithms. Zhu et al. [16] proposed an improved hybrid algorithm that combines the global exploration capability of BKA with the local exploitation characteristics of PSO, while introducing the differential mutation operator from differential evolution (DE) to enhance population diversity and prevent premature convergence. Li et al. [17] addressed the insufficient accuracy of BKA in solving practical problems by proposing an improved version that integrates the OOA with vertical and horizontal crossover enhancements, effectively improving the algorithm’s performance. To more clearly compare the performance differences among various algorithms, we present the comparison in Table 1.

Zhang et al. [18] proposed a PIO–L-BFGS hybrid strategy, which combines the pigeon-inspired optimization (PIO) algorithm with the limited-memory Broyden–Fletcher–Goldfarb–Shanno (L-BFGS) algorithm [19], effectively improving local search accuracy and convergence performance. Cuevas et al. [20] introduced a cheetah optimizer hybrid approach based on opposition-based learning and diversity metrics (CO-DO). In this approach, an opposition-based learning (OBL) mechanism is incorporated into the Cheetah Optimizer (CO), where candidate solutions are updated by considering their counterparts in opposite regions of the search space, thereby expanding the search range and enhancing population diversity. In addition, the BHJO algorithm proposed by Zitouni et al. [21] also integrates an OBL strategy within a hybrid metaheuristic framework, improving the global exploration capability of the algorithm and further validating the effectiveness of OBL in expanding the search space. These studies collectively indicate that quasi-Newton-based refinement and OBL mechanisms are effective in enhancing local exploitation accuracy and global exploration capability, respectively.

Despite the fact that the aforementioned improvements have achieved certain success in enhancing the performance of the BKA, issues such as insufficient global exploration capability and inadequate local exploitation accuracy still persist. To address these problems, this paper proposes a black-winged kite algorithm integrating opposition-based learning and quasi-Newton strategy (OQBKA) to further improve the overall optimization performance of the algorithm. Specifically, a mirror imaging strategy based on convex lens imaging (MOBL) [22] is introduced during the migration phase to generate dynamic opposite solutions, thereby enhancing global search capability and effectively improving population diversity. In the later stage of iteration, the L-BFGS algorithm with boundary constraints is integrated to perform local optimization on the top five individuals with the best fitness, thereby strengthening the algorithm’s local exploitation ability.

To verify the effectiveness of the integrated strategies, ablation experiments were conducted on the CEC2017 benchmark function set to analyze the impact of the MOBL and L-BFGS strategies on algorithm performance. In addition, comparative experiments on the CEC2022 benchmark set demonstrate that the proposed OQBKA algorithm significantly outperforms the original BKA in terms of convergence speed and optimization accuracy. Furthermore, to evaluate its potential for practical engineering applications, the OQBKA was applied to several constrained engineering optimization problems, including the step-cone pulley optimization problem [23], corrugated bulkhead design [24], and reactor network design [25]. The experimental results confirm the applicability of OQBKA in solving real-world constrained engineering optimization problems.

The main contributions of this study are as follows:

(1) The novel integration of MOBL and L-BFGS into BKA, creating a more balanced and powerful optimizer;
(2) A comprehensive empirical validation using standardized benchmarks, showing significant improvements in convergence speed and solution accuracy;
(3) The demonstration of OQBKA’s effectiveness in solving complex, constrained real-world engineering problems.

## 2. Materials and Methods

### 2.1. Review of BKA

The black-winged kite exhibits distinctive migratory and predatory behaviors, characterized by long-distance movement and precise hovering during hunting. Drawing inspiration from these biological characteristics, Wang et al. [11] developed the BKA, which models these behaviors through three main computational components.

(1) Population Initialization

In the BKA, the first step of population initialization is to generate a set of random solutions. The position of each black-winged kite in the population can be represented by the following matrix:

(1) $$BK=\left[\begin{array}{cccc} BK_{1,1} & BK_{1,2} & \ldots & BK_{1,\dim}\\ BK_{2,1} & BK_{2,2} & \ldots & BK_{2,\dim}\\ \ldots & \ldots & \ddots & \vdots \\ BK_{pop,1} & BK_{pop,2} & \ldots & BK_{pop,\dim} \end{array}\right]$$

where pop denotes the population size, dim represents the problem dimension, and $BK_{i,j}$ indicates the position of the $i^{th}$ black-winged kite in the $j^{th}$ dimension.

(2) $$X_{i}=BK_{lb}+r\times \left(BK_{ub}-BK_{lb}\right)$$

During the initialization process, the BKA selects the individuals with the best fitness values, denoted as $X_{L}$, from the initial population as leaders. The mathematical expression is given as follows:

(3) $$f_{best}=\min(f(X_{i})),$$

(4) $$X_{L}=X\left(find\left(f_{best}==f\left(X_{i}\right)\right)\right)$$

where $f_{best}$ denotes the optimal fitness value in the population, $X_{i}$ represents the $i^{th}$ individual in the population, and find refers to the search operation used to locate individuals that meet specific conditions.

(2) Attack Behavior

As predators, black-winged kites adjust the angles of their wings and tails according to wind speed, hovering in the air to observe their prey before swiftly diving at the appropriate moment to capture it. In the algorithm, the predation strategy is modeled as attack behaviors with distinct characteristics, corresponding to the global search and local search processes, respectively. The mathematical model can be expressed as follows:

(5) $$y_{t+1}^{i,j}=\left\{\begin{array}{l} y_{t}^{i,j}+n\times (1+\sin(r))\times y_{t}^{i,j},\;if\;p<r\\ y_{t}^{i,j}+n\times (2r-1)\times y_{t}^{i,j},\;else \end{array}\right.$$

(6) $$n=0.05\times e^{-2\times {\left(\frac{t}{T}\right)}^{2}}$$

where $y_{t}^{i,j}$ and $y_{t+1}^{i,j}$ represent the positions of the $i^{th}$ black-winged kite in the $j^{th}$ dimension at the $t^{th}$ and ${\left(t+1\right)}^{th}$ iterations, respectively; n denotes a parameter that varies with the number of iterations and is used to adjust the step size; p is a constant with a value of 0.9; t represents the current iteration number, and T denotes the maximum number of iterations.

(3) Migration Behavior

Bird migration is a complex collective behavior that is typically influenced by environmental factors such as climate change and food availability. Inspired by this phenomenon, the BKA designs a dynamic leader migration strategy. The basic idea is as follows: if the current leader’s fitness value is inferior to that of a randomly selected candidate individual, the leader relinquishes its leadership and joins the migrating population. Conversely, when the leader’s fitness value is superior to that of the random individual, it continues to guide the population toward the target region. This strategy allows for the dynamic selection of high-quality leaders, thereby ensuring successful migration. The mathematical model of the migration behavior is as follows:

(7) $$y_{t+1}^{i,j}=\left\{\begin{array}{l} y_{t}^{i,j}+C\left(0,1\right)\times (y_{t}^{i,j}-L_{t}^{j}),\;if\;F_{i}<F_{ri}\\ y_{t}^{i,j}+C\left(0,1\right)\times (L_{t}^{j}-m\times y_{t}^{i,j}),\;else \end{array}\right.$$

(8) $$m=2\times \sin(r+\frac{\pi }{2})$$

where $L_{t}^{j}$ denotes the leadership score of the black-winged kite in the $j^{th}$ dimension at the $t^{th}$ iteration; $y_{t}^{i,j}$ and $y_{t+1}^{i,j}$ represent the positions of the $i^{th}$ black-winged kite in the $j^{th}$ dimension at the $t^{th}$ and ${\left(t+1\right)}^{th}$ iterations, respectively; $F_{i}$ denotes the current position of any black-winged kite in the $j^{th}$ dimension at iteration $t^{th}$; $F_{ri}$ represents the fitness value of a randomly selected position in the $j^{th}$ dimension obtained from any black-winged kite at iteration $t^{th}$; and $C\left(0,1\right)$ is a random variable following the Cauchy distribution, whose probability density function is defined as follows:

(9) $$f(x,\delta ,\mu )=\frac{1}{\pi }\frac{\delta }{\delta^{2}+{\left(x-\mu \right)}^{2}},-\infty <x<+\infty$$

where x denotes the random variable, δ is the scale parameter that controls the width of the distribution, and μ represents the location parameter.

When δ=1 and μ=0, it simplifies to the standard Cauchy distribution:

(10) $$f(x,\delta ,\mu )=\frac{1}{\pi }\frac{1}{1+x^{2}},-\infty <x<+\infty$$

### 2.2. MOBL

Opposition-based learning (OBL) is an optimization strategy that expands the search range by generating opposite solutions based on the current ones, effectively enhancing population diversity and improving global search capability. In the case of BKA, its global search ability tends to decline in the later stages of the search process, leading to reduced population diversity and a higher risk of trapping in local optima. Although the algorithm incorporates Cauchy mutation to increase perturbation amplitude, its effect is limited when dealing with high-dimensional multimodal functions, making it difficult to significantly improve search performance. Therefore, a stronger exploration mechanism needs to be introduced in the later stages of the algorithm to prevent premature convergence.

In this study, the MOBL method proposed by Yao et al. [18] is adopted. The core idea of this approach is to simulate the physical principle of convex lens imaging, enabling the opposite solutions to be uniformly distributed within the search space, thereby improving search efficiency. Specifically, for a current solution X, its opposite solution $X^{\prime }$ is calculated as follows:

(11) $$X^{\prime }=\frac{ub+lb}{2}+\frac{ub+lb}{2q}-\frac{X}{q}$$

where ub and lb denote the upper and lower bounds of the search space, respectively; q is the mirror factor, calculated as $q=10\times \left(1-2\times {\left(t/T\right)}^{2}\right)$; T represents the total number of iterations, and t is the current iteration number.

Compared with the standard OBL strategy, the MOBL strategy can generate higher-quality opposite solutions within a broader search space, effectively enhancing the global exploration capability of the population.

By introducing the MOBL strategy after the attacking behavior and migration behavior of the BKA, population diversity can be effectively improved, helping the algorithm escape from local optima. Meanwhile, the mirror factor in MOBL gradually decreases with the number of iterations, leading to a convergence in the amplitude of the generated opposite solutions. This facilitates more refined local search and convergence control in the later stages of iteration, thereby achieving a balanced trade-off between global exploration and local exploitation capabilities.

### 2.3. L-BFGS

The Newton method is a classical unconstrained optimization approach with favorable convergence properties. Its core idea is to compute the hessian matrix of the objective function to obtain a more accurate search direction. However, since each iteration requires the computation and solution of the hessian matrix, the computational cost is high, which limits its applicability to high-dimensional problems. To reduce computational complexity, the quasi-Newton method [26] was proposed. The quasi-Newton method iteratively constructs a symmetric positive definite matrix to approximate the inverse of the hessian matrix of the objective function, thereby significantly reducing computational and storage overhead.

The Broyden–Fletcher–Goldfarb–Shanno (BFGS) algorithm [27] is one of the most representative methods among the quasi-Newton family. In each iteration, it dynamically updates an approximation of the inverse hessian matrix using gradient information to determine the descent direction. To further reduce storage and computational overhead, the limited-memory BFGS (L-BFGS) method retains only a limited number of historical information vectors and implicitly approximates the inverse hessian matrix, making it particularly suitable for large-scale optimization problems. The updating formula for the inverse hessian matrix is as follows:

(12) $$H_{k+1}=(I-\rho_{k}s_{k}y_{k}^{T})H_{k}(I-\rho_{k}y_{k}s_{k}^{T})+\rho_{k}s_{k}s_{k}^{T}$$

where $H_{k}$ denotes the approximate inverse hessian matrix at the $k^{th}$ iteration, $s_{k}=x_{k+1}-x_{k}$ represents the position change vector, $y_{k}=\nabla f_{k+1}-\nabla f_{k}$ denotes the gradient change vector, and $\rho_{k}=1/y_{k}^{T}s_{k}$ is the scaling factor. This formula is used to update the approximation of the inverse hessian matrix, thereby obtaining a new search direction and accelerating the convergence process.

In the original version of the BKA, the leader update strategy retains only the individual with the best current fitness, which may cause the algorithm to become trapped in local optima, reduce population diversity, and eventually lead to premature convergence. To enhance the local exploitation capability and improve the solution quality, this study introduces the L-BFGS algorithm in the later stages of iteration to perform refined local searches on several high-quality individuals.

To prevent local search from interfering prematurely with global exploration, this strategy is activated during the later stages of algorithm iteration. Once the triggering condition is met, the top K individuals with the best fitness values in the current population are selected, and each of their current positions is used as an initial point to invoke the objective function and perform local refinement using the L-BFGS algorithm. Since L-BFGS is a local optimization method with relatively high computational cost, it is not suitable to be applied to the entire population. In this work, L-BFGS is only performed on the top 5 individuals with the best fitness values. This elitist strategy ensures that local refinement is conducted in promising regions of the search space, thereby improving solution quality while significantly reducing computational overhead. Experimental analysis indicates that a larger number of refined individuals can improve search accuracy but significantly increases computational cost and reduces efficiency; conversely, a smaller number may lead to insufficient local exploitation, degrading solution precision. Therefore, K is set to 5 to balance effectiveness and efficiency. Additionally, the maximum number of L-BFGS iterations is set to 20, and the function tolerance is fixed at $10^{-6}$. Boundary constraints are handled by projecting any infeasible solutions back into the predefined search space. During the local search process, if the optimized result outperforms the original individual, the individual’s position and fitness value are updated with the new solution; otherwise, the original solution is retained. By incorporating this strategy, the algorithm maintains strong global exploration capability while performing deeper exploitation of high-quality solutions, effectively enhancing overall optimization accuracy and convergence performance.

### 2.4. OQBKA

The specific implementation steps of the proposed OQBKA are presented in Algorithm 1.
**Algorithm 1:** OQBKA
**Input**: Maximum number of iterations
T, population size
N, lower and upper bounds of the search space
lb and
ub, problem dimension
dim, and fitness function
fobj. **Output**: Best solution
$X_{best}$ and its fitness
$F_{best}$.
1.Initialize the population $X_{i}(i=1,2,\ldots ,N)$ randomly within [lb,ub].2.Evaluate the fitness $F_{i}=f(X_{i})$ of each black-winged kite.3.Set iteration counter t=1.4.**while (**t≤T**) do**5. Sort the population according to fitness values.6. Set the best individual as $X_{L}$.7./* Attacking behavior */8. **for** i=1 to T **do**9.    Compute the attack factor n using Equation (6).10.  Generate random numbers p,r∈(0,1).11.  **if** p<r **then**12.   
$X_{i}^{t+1}=X_{i}^{t}+n\times (1+\sin(r))\times X_{i}^{t}$13.  **else**14.   
$X_{i}^{t+1}=X_{i}^{t}+n\times (2r-1)\times X_{i}^{t}$15.  Apply boundary control.16.  Evaluate the new solution and keep the better one.17./* Migration behavior */18.  Compute the migration factor m using Equation (8).19.  **if** $F_{i}<F_{ri}$ **do**20.   
$X_{i}^{t+1}=X_{i}^{t}+C\left(0,1\right)\times (X_{i}^{t}-X_{L})$21.  **else**22.   
$X_{i}^{t+1}=X_{i}^{t}+C\left(0,1\right)\times (X_{L}-m\times X_{i}^{t})$23.  Apply boundary control.24.  Evaluate the new solution and keep the better one.25./* MOBL */26.  Compute the mirror factor q using the formula $q=10\times \left(1-2\times {\left(t/T\right)}^{2}\right)$.27.  Compute the opposite solution $X^{\prime }$ using Equation (11).28.  Apply boundary control.29.  Evaluate the new solution and keep the better one.30. **end for**31./* L-BFGS */32. **if** t≥0.7T **then**33.  Select the top K=min(5,pop) elite individuals.34.  Apply L-BFGS local search to each elite solution.35.  Replace the original solution if improvement is achieved.36. **end if**37.  Update global best solution $X_{best}$ and $F_{best}$.38. Set t=t+1.39.**end while**40.**return** $X_{best}$ and $F_{best}$.

**Figure 1:**
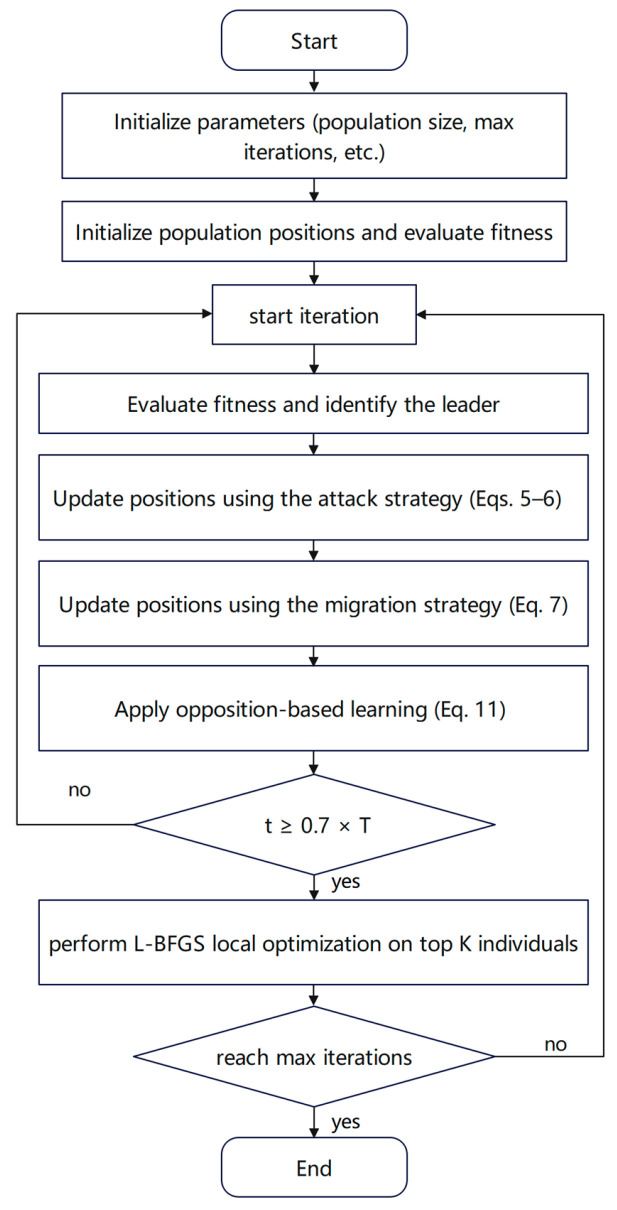
Algorithm flowchart.

The source code of OQBKA (commit 46442f3) is publicly available on GitHub at https://github.com/saturnus120/OQBKA (accessed on 25 December 2025). Figure 1 shows the flowchat of OQBKA.

Although rigorous global convergence proofs for metaheuristic algorithms remain challenging, OQBKA effectively enhances its convergence performance through several key mechanisms. First, the algorithm inherits the elitism mechanism from BKA, ensuring that the best solution found so far is never lost during the iterative process. Second, the MOBL strategy strengthens global exploration and effectively mitigates premature convergence. Furthermore, the integration of the L-BFGS method in the late stage of optimization leverages its well-established local convergence guarantees under the assumption of objective function smoothness, significantly improving solution accuracy. These improvements in convergence behavior, however, could potentially increase computational cost. To verify that OQBKA remains efficient, we analyze its time complexity per iteration. Let N denote the population size and D the problem dimensionality. The main computational cost of OQBKA arises from the attack, migration, and mirror-based opposition learning operations during population evolution. In each generation, these operators update N individuals of dimensionality D, resulting in a per-iteration complexity of O(ND). The L-BFGS local search is applied only to the top 5 individuals during the final 30% of iterations, introducing an additional overhead of O(TD) (constant factors omitted). Consequently, the overall time complexity of OQBKA is O(TND), which is on the same order as the original BKA and does not impose a significant computational burden.

## 3. Results

### 3.1. Experiment Setup

The experimental setup of this study consists of four parts:

(1) Perform parameter sensitivity experiments on selected CEC2022 benchmark functions to determine optimal algorithm parameter settings.
(2) Ablation experiments are conducted on the CEC2017 benchmark set to analyze the effectiveness of the introduced improvement strategies.
(3) Comparative experiments are performed on the CEC2022 standard benchmark function set against other swarm intelligence algorithms to comprehensively evaluate the overall performance of the proposed improved algorithm.
(4) Perform population diversity experiments on unimodal and multimodal functions to evaluate the algorithm’s exploration capability.
(5) Engineering design experiments are conducted on three constrained optimization problems to evaluate the applicability and robustness of the proposed algorithm, where general nonlinear constraints are handled using a penalty-based objective function that penalizes infeasible solutions by adding a large constant to their fitness values, effectively guiding the search toward the feasible region.

### 3.2. Parameter Sensitivity Analysis

Among the key parameters in OQBKA, the L-BFGS activation threshold k (i.e., L-BFGS is triggered when t≥kT) has a significant impact on the balance between exploration and exploitation. To determine its optimal value, we evaluate $k\in \left\{0.1,\;0.4,\;0.7,\;0.9\right\}$ on selected CEC2022 benchmark functions (F1, F4, F7, F10) in 10 dimensions over 30 independent runs. These functions represent four distinct types of optimization landscapes: the unimodal F1, the multimodal F4, the hybrid F7, and the composition function F10, ensuring a comprehensive evaluation. As summarized in Table 2, k=0.7 consistently achieves the best performance in terms of solution accuracy and convergence stability. Smaller values lead to premature convergence due to early local search, while larger values delay refinement and result in suboptimal final solutions. Therefore, k=0.7 is adopted in the final algorithm.

In addition, the number of elite individuals refined by the L-BFGS local search is set to K=5, as a larger value would lead to significantly higher computational overhead, while a smaller value may result in insufficient local exploitation and premature convergence. The scaling factor q in the MOBL strategy adopts the original formulation $q=10\times \left(1-2\times {\left(t/T\right)}^{2}\right)$, which has been validated in the original study and confirmed effective by our preliminary experiments.

### 3.3. Ablation Experiments on CEC2017

To evaluate the effectiveness of each improvement strategy, ablation experiments are conducted on 29 benchmark functions from the CEC2017 test suite. Among these functions, F1–F3 are unimodal functions mainly used to test the convergence accuracy of the algorithm; F4–F11 are multimodal functions designed to assess the algorithm’s global search capability in escaping local optima; F12–F20 are hybrid functions, and F21–F29 are composition functions with higher overall complexity, which are employed to comprehensively evaluate the algorithm’s adaptability to complex optimization problems.

Based on the original BKA, two variants were developed by separately incorporating the L-BFGS and MOBL strategies. The algorithm integrated with the L-BFGS strategy is denoted as LBKA, while the one incorporating the MOBL strategy is denoted as OBKA. These two variants, along with the OQBKA and the original BKA, are compared in this experiment. By statistically analyzing the mean fitness values, standard deviations, and average rankings of each algorithm across all benchmark functions, the contribution of each strategy to the overall performance is evaluated.

**Figure 2:**
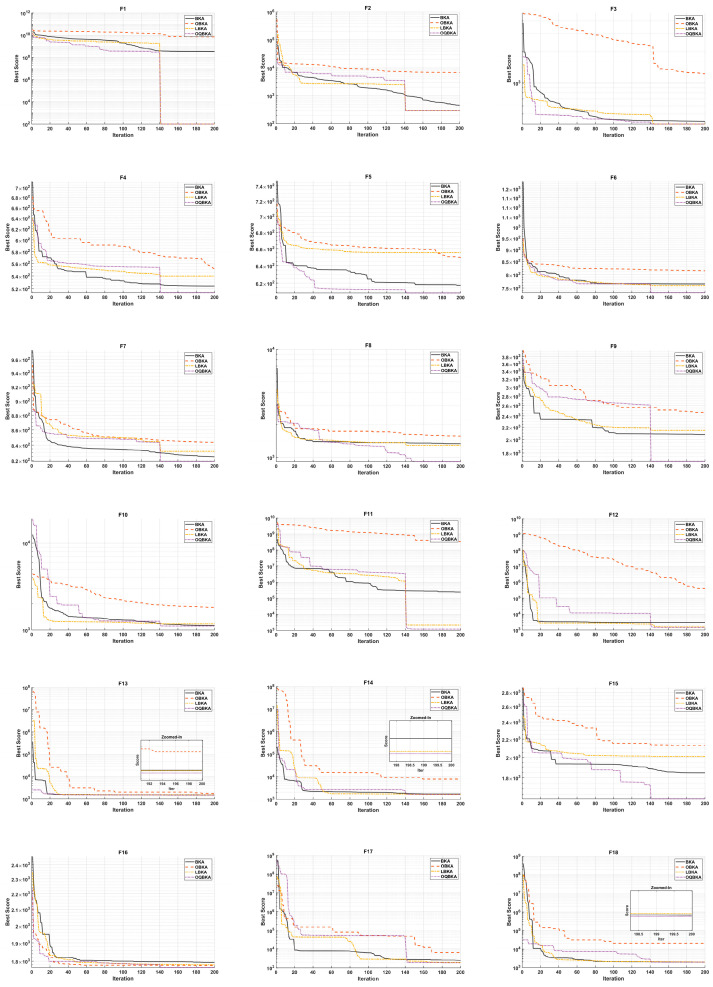

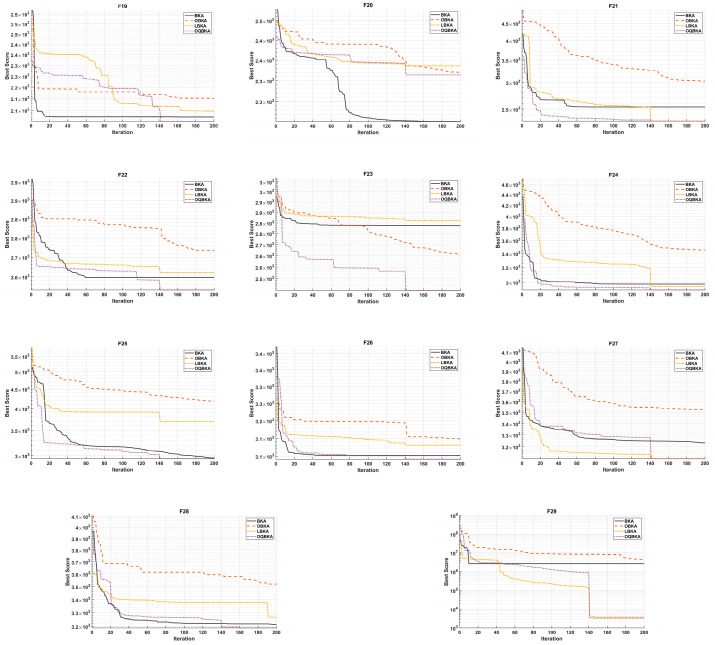
Convergence curves of CEC2017 test functions.

All experiments were conducted strictly following the parameter settings recommended in the original papers of each algorithm. The maximum number of iterations was uniformly set to 200, and each experiment was independently run 30 times to ensure the reliability and statistical validity of the results. The statistical outcomes are summarized in Table 3, where bold values indicate the best results, and the convergence behavior of the algorithms is illustrated in Figure 2.

According to the experimental results, the following conclusions can be drawn:

(1) For complex functions such as F5, F6, F8, F9, F11, F14, F17, and F18, OBKA performs significantly better than BKA, indicating that the incorporation of the MOBL strategy effectively enhances the algorithm’s global search capability, particularly when dealing with complex multimodal functions.
(2) LBKA achieves a substantially better average ranking than BKA and obtains optimal or near-optimal results on functions F1, F3, F11, F12, F14, F17, and F18, demonstrating that the quasi-Newton strategy improves the algorithm’s local search efficiency and convergence accuracy, thereby strengthening its ability to locate high-quality solutions.
(3) The OQBKA algorithm, which integrates both improvement strategies, demonstrates overall performance that far surpasses either variant using a single strategy. It achieves the best results on 20 benchmark functions, with an average ranking of 1.34, significantly outperforming OBKA, LBKA, and the original BKAs. Specifically, OQBKA and LBKA exhibit comparable performance on the unimodal functions F1–F3, both clearly outperforming the other algorithms; OQBKA achieves the best performance on six out of seven multimodal benchmark functions F4–F10; it ranks first on seven out of ten hybrid functions F11–F20, showing a distinct advantage particularly on F14–F19; and it obtains the best results on seven out of nine composite functions F21–F29.These findings indicate that the integration of the two strategies produces a complementary effect: the MOBL strategy enhances the algorithm’s ability to explore a broader solution space, while the L-BFGS strategy improves solution accuracy and convergence speed. As a result, OQBKA demonstrates superior optimization performance across various types of benchmark functions, particularly excelling in solving multimodal and complex optimization problems.
(4) In terms of standard deviation, OQBKA exhibits strong stability across most functions. Notably, for F7, F15, and F25, the standard deviations are exceptionally small, indicating that the algorithm produces stable and reliable results.

### 3.4. Comparison Experiments on CEC2022

The CEC2022 test suite encompasses a variety of complex optimization problems and is widely used for standardized evaluation of swarm intelligence algorithms. The suite contains 12 functions, which can be categorized into four types based on their characteristics: F1–F2 are unimodal functions used to test the algorithm’s local search accuracy and convergence speed; F3–F5 are multimodal functions designed to assess the algorithm’s ability to escape local optima; F6–F8 are hybrid functions that combine multiple basic functions to increase problem complexity and evaluate the algorithm’s adaptability in non-uniform search spaces; F9–F12 are composition functions in which multiple functions with different characteristics are nested and integrated, providing a comprehensive assessment of the algorithm’s global search capability and robustness.

On the CEC2022 test suite, the proposed OQBKA algorithm is evaluated against the original BKA and several mainstream swarm intelligence algorithms. The comparison algorithms include four classical swarm intelligence algorithms: PSO, HHO, GWO, and SSA; three recently proposed algorithms: ALA, MSO, and WMA; and two improved variants of BKA: SCBKA and IBKA. In addition, to verify the statistical significance of the results, the performance of each algorithm on the test functions is analyzed using the Wilcoxon rank-sum test.

All algorithms are compared under a unified parameter configuration: N for population size and T=200 for maximum number of iterations. To ensure statistical reliability, each algorithm is independently executed 30 times on the 12 test functions, with the best fitness value recorded for each run, and the global best results highlighted in bold. The experimental results are presented in Table 4 and Table 5, showing the performance of OQBKA compared with the 10 benchmark algorithms on the CEC2022 test suite.

To comprehensively evaluate algorithm performance, a multi-dimensional evaluation metric system is employed, including:

(1) Statistical significance test: The Wilcoxon rank-sum test (significance level α=0.05) is used to evaluate differences in algorithm performance. In the results, the symbols “−”, “=“, and “+” indicate that a comparison algorithm is statistically significantly worse than, equivalent to, or significantly better than OQBKA, respectively.
(2) Solution ranking: Algorithms are ranked based on their average fitness values on the test functions, with lower fitness values corresponding to higher ranks. When average fitness values are equal, the algorithm with the smaller standard deviation receives a higher rank, providing a comprehensive reflection of solution quality and algorithm stability. Moreover, the Wilcoxon test is not involved in the ranking process. It is used solely for post hoc analysis to assess whether the observed performance differences are statistically significant.
(3) Convergence efficiency analysis: The convergence curves are used to compare the dynamic optimization capabilities of the algorithms throughout the iteration process.

**Figure 3:**
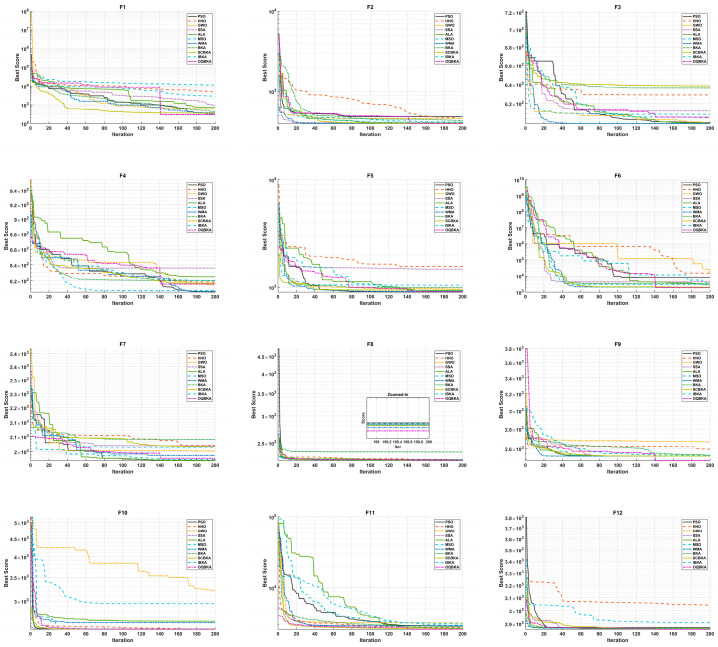
Convergence curves of CEC2022 test functions (dim = 10).

As shown in Table 4, which presents the experimental results on the 10-dimensional test functions, OQBKA achieves the best rankings on eight functions: F1, F2, F6, F8, F9, F10, F11, and F12, with an overall average ranking of 2.5. Although it does not obtain the best fitness value on F10, the Wilcoxon rank-sum test indicates that the difference between OQBKA and the best-performing algorithm is not statistically significant. On F1, F2, and F11, OQBKA successfully converges to the theoretical global optimum, demonstrating its precise search capability in high-dimensional complex spaces. Convergence curve analysis shows that OQBKA exhibits the fastest convergence speed on all functions except F3–F5. In particular, for functions F6–F12, the slopes of OQBKA’s convergence curves are significantly steeper than those of the comparison algorithms, indicating that the integrated strategies effectively accelerate the population’s convergence toward the optimal regions. To further illustrate the convergence behavior, Figure 3 shows the detailed convergence curves of OQBKA. The algorithm exhibits a distinct “two-stage decline” pattern: during the early iterations, the MOBL strategy enables it to rapidly reduce the fitness value, achieving efficient global exploration; in the middle and later stages, as the mirror factor gradually converges and triggers the L-BFGS local search, OQBKA maintains a slow yet steady downward trend, demonstrating strong local exploitation and continuous convergence capability. This pattern indicates that OQBKA achieves a good dynamic balance between exploration and exploitation

Table 5 presents the experimental results of different algorithms on 20-dimensional optimization problems. The results show that OQBKA achieves the best rankings on eight test functions. Although it performs slightly worse than PSO on F7, its best fitness value is close to that of PSO, remaining highly competitive. Furthermore, as the problem dimensionality increases, OQBKA maintains a leading advantage, particularly demonstrating excellent optimization capability on unimodal functions and complex composition functions.

**Figure 4:**
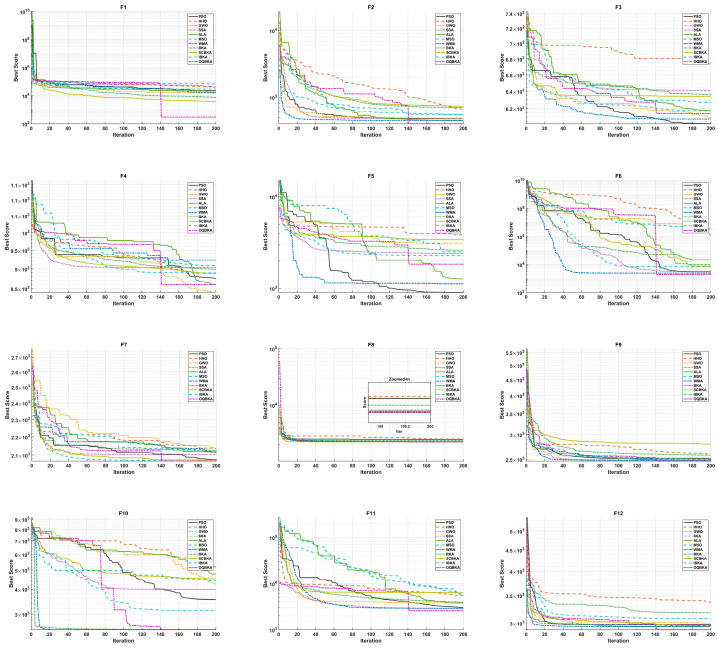
Convergence curves of CEC2022 test functions (dim = 20).

In terms of stability, OQBKA achieves the lowest standard deviation on more than half of the test functions, indicating that its optimization results are more consistent and can maintain high reproducibility across multiple independent runs. As shown by the convergence curves in Figure 4, OQBKA demonstrates faster convergence on all test functions except F3–F5, with slopes significantly steeper than those of the comparison algorithms, further confirming its efficient global search and local exploitation capabilities.

Moreover, the convergence curves of OQBKA exhibit a distinct “two-stage descent” pattern: in the early iterations, the MOBL strategy enables the algorithm to rapidly reduce the fitness value, achieving efficient global exploration; in the middle-to-late stages, as the mirror factor gradually converges and triggers the L-BFGS local search, the algorithm maintains a slow yet steady descent, demonstrating strong local exploitation and sustained convergence capability. This behavior indicates that OQBKA achieves a well-balanced dynamic trade-off between exploration and exploitation.

In terms of statistical significance analysis, the Wilcoxon rank-sum test results indicate that OQBKA demonstrates a significant advantage over the other algorithms on most test functions. In particular, for functions F1, F2, F6, and F8–F12, OQBKA performs significantly better than the comparison algorithms, clearly highlighting its superior capability in handling high-dimensional complex optimization problems.

### 3.5. Search Dynamics Visualization

To quantitatively analyze the impact of the proposed enhancement strategies on population diversity and convergence behavior, comparative experiments are conducted on representative unimodal and multimodal benchmark functions. The diversity curves and convergence trajectories of BKA and OQBKA are presented for analysis.

**Figure 5:**
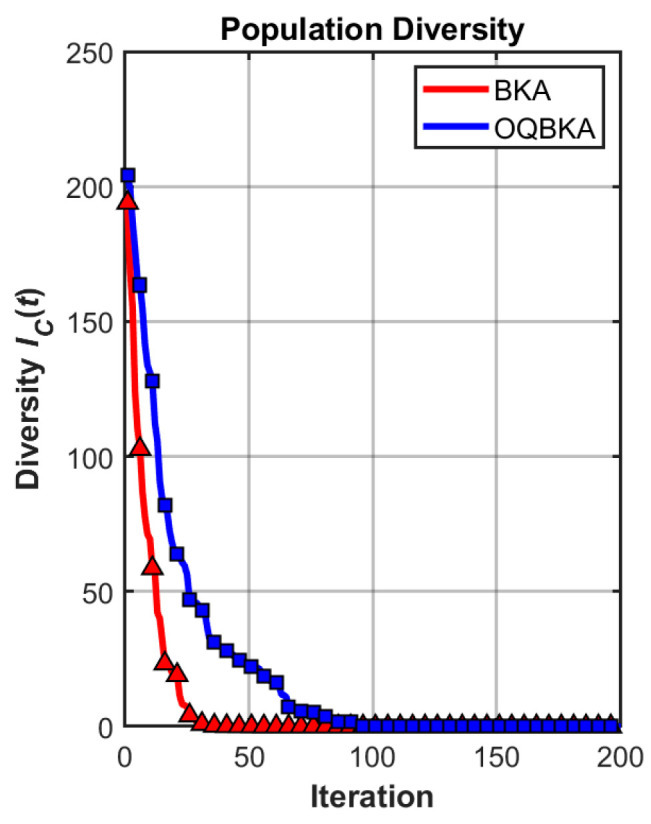
Population diversity curve on unimodal functions.

**Figure 6:**
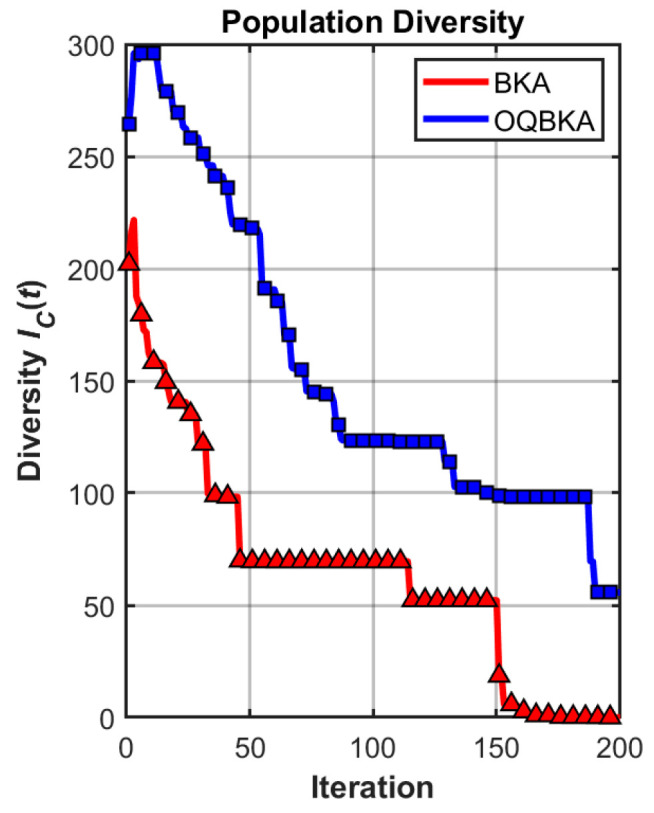
Population diversity curve on multimodal functions.

As shown in Figure 5 and Figure 6, OQBKA consistently maintains significantly higher population diversity than BKA throughout the optimization process. The diversity metric adopted in this study, denoted as $D_{c}(t)$, is defined as the average Euclidean distance among individuals in the population and is used to characterize the spatial distribution of the population. This metric remains at a relatively high level during the middle and late stages of OQBKA, indicating that the incorporation of the MOBL strategy effectively suppresses premature convergence and preserves the global exploration capability of the algorithm.

**Figure 7:**
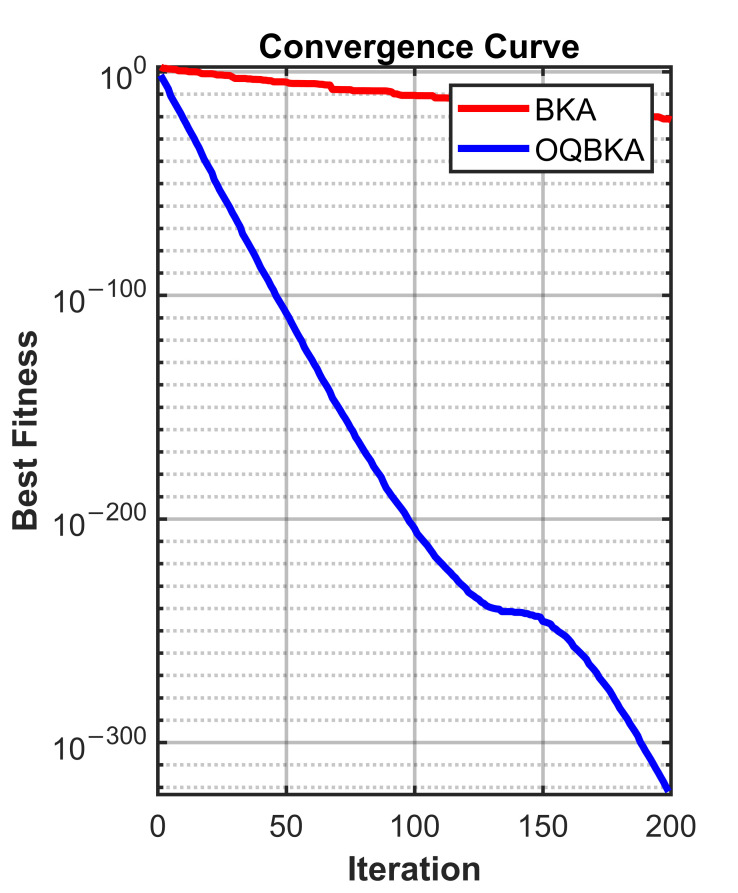
Convergence curve on unimodal functions.

**Figure 8:**
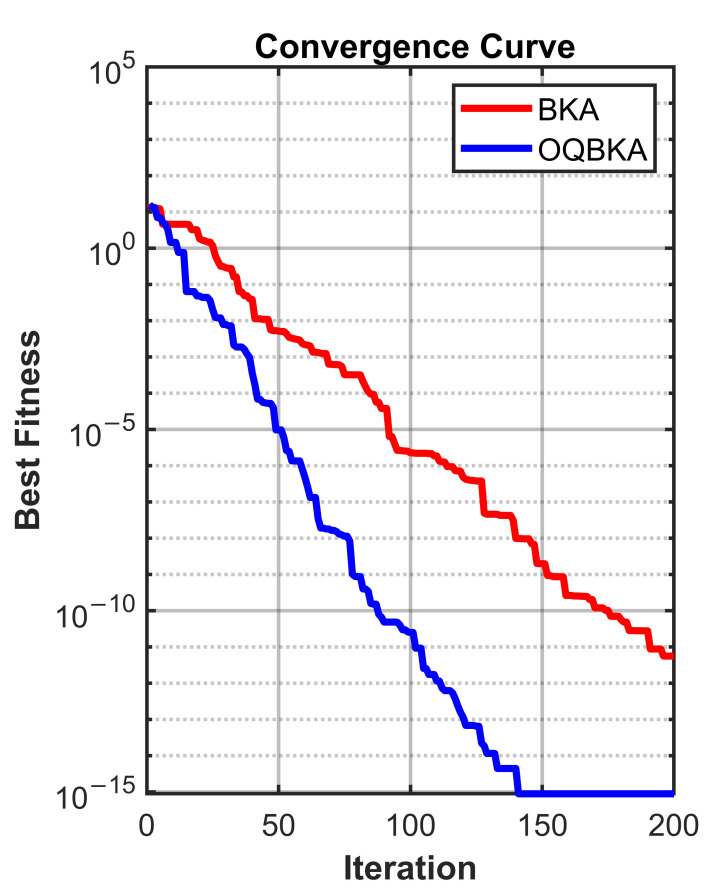
Convergence curve on multimodal functions.

In contrast, the population diversity of BKA decreases rapidly after approximately 50 iterations, revealing an evident early stagnation phenomenon. This issue becomes more pronounced in the multimodal case, as shown in Figure 6, where the diversity of BKA collapses sharply around iteration 150, whereas OQBKA is able to maintain a relatively stable diversity level until convergence.The corresponding convergence curves, as shown in Figure 7 and Figure 8, further demonstrate that higher population diversity can be translated into superior solution quality and more stable convergence behavior. OQBKA exhibits a smoother convergence process and achieves lower final fitness values than BKA on both types of functions. In particular, in the multimodal scenario, BKA tends to be trapped in local optima, while OQBKA continues to improve and ultimately approaches the global optimum.

### 3.6. Engineering Design Problems

For general nonlinear constraints in engineering problems, a penalty-based objective function is adopted: infeasible solutions are penalized by adding a large constant to their fitness value, effectively guiding the search toward the feasible region.

(1) Step-Cone Pulley Problem

A step-cone pulley is a stepped conical structure composed of a series of pulleys. They are used in pairs to change the speed ratio between shafts. Power is transmitted from one shaft to another distant shaft by a belt or rope running over the pulleys. The primary objective of this problem is to minimize the weight of a four-step conical pulley using five design variables: four variables corresponding to the diameters of each step and a fifth variable representing the pulley width. The problem includes 11 nonlinear constraints to ensure that the transmitted power equals 0.75 hp. The mathematical formulation of the problem is defined as follows:

(13) $$W=\frac{\pi }{4}\rho w\left[d_{1}^{2}\left\{1+{\left(\frac{N_{1}}{N}\right)}^{2}\right\}+d_{2}^{2}\left\{1+{\left(\frac{N_{2}}{N}\right)}^{2}\right\}+d_{3}^{2}\left\{1+{\left(\frac{N_{3}}{N}\right)}^{2}\right\}+d_{4}^{2}\left\{1+{\left(\frac{N_{4}}{N}\right)}^{2}\right\}\right]$$

where W denotes the weight of the four-step step-cone pulley; $\rho =7200\;kg/m^{3}$ denotes the material density; w denotes the radial width of the pulley, with range 16 mm≤w≤100 mm; $d_{i}$ denotes the diameter of the $i^{th}$ pulley, with range $40\;mm\leq d_{i}\leq 100\;mm$; N is the input rotational speed; and $N_{i}$ is the output rotational speed of the $i^{th}$ pulley.

The constraints are as follows:

(14) $$h_{1}\left(x\right)=c_{1}-c_{2}=0$$

(15) $$h_{2}\left(x\right)=c_{1}-c_{3}=0$$

(16) $$h_{3}\left(x\right)=c_{1}-c_{4}=0$$

(17) $$g_{1,2,3,4}\left(x\right)=R_{i}-2\geq 0;\;i=1,\ldots ,4$$

(18) $$g_{5,6,7,8}\left(x\right)=P_{i}-\left(0.75\times 745.6998\right)\geq 0;\;i=1,\ldots ,4$$

(19) $$c_{i}=\frac{d_{i}\pi }{4}\left(\frac{N_{i}}{N}+1\right)+\frac{d_{i}^{2}{\left(\frac{N_{i}}{N}-1\right)}^{2}}{4a}+2a$$

(20) $$P_{i}=stw\left(1-P_{i}\right)\times \left(\frac{d_{i}\pi N_{i}}{60}\right);\;i=1,\ldots ,4$$

where $h_{1}\left(x\right)$, $h_{2}\left(x\right)$, $h_{3}\left(x\right)$ represent the nonlinear equality constraints; $g_{i}\left(x\right)$ represents the nonlinear inequality constraints; $c_{i}$ is the belt length of the $i^{th}$ pulley; $R_{i}$ is the tension on the $i^{th}$ pulley; $P_{i}$ is the power transmitted to the $i^{th}$ pulley; a is the center distance of the pulleys, representing the distance between the centers of the two pulleys, with a value of 3 m; s is the permissible material strength, with a value of 1.75 MPa; t is the axial thickness of the pulley, with a value of 8 mm; μ is the coefficient of dynamic friction, with a value of 0.35.

**Figure 9:**
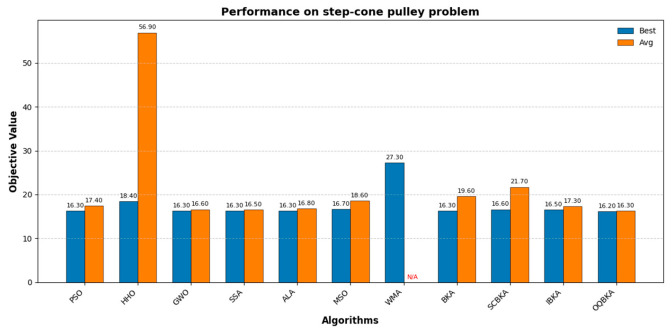
Performance on step-cone pulley problem.

Table 6 and Figure 9 presents the performance of 11 algorithms in solving the step-cone pulley problem. Each algorithm was independently executed 20 times, and the best value, worst value, standard deviation, and mean value were recorded. It can be observed that the OQBKA algorithm achieved the best results among all algorithms across the 20 independent runs, significantly outperforming the other compared methods. The extremely small standard deviation indicates that OQBKA exhibits high stability with minimal result fluctuations across multiple runs. Moreover, its worst performance also remains at a relatively high level, fully demonstrating the superior optimization accuracy, stability, and robustness of the OQBKA algorithm.

(2) Corrugated Bulkhead Design

The corrugated bulkhead design problem involves four design variables, denoted as $x_{1}$, $x_{2}$, $x_{3}$ and $x_{4}$. The optimization objective is to minimize the weight of the corrugated bulkhead of the tanker. The mathematical model for this problem is as follows:

Objective function:

(21) $$f\left(x\right)=\frac{5.885x_{4}(x_{1}+x_{3})}{x_{1}+{\left(x_{3}^{2}+x_{2}^{2}\right)}^{1/2}}$$

The constraints are as follows:

(22) $$g_{1}\left(x\right)=x_{2}x_{4}\left(0.4x_{1}+\frac{1}{6}x_{3}\right)-0.894\left[x_{1}+{\left(x_{3}^{2}-x_{2}^{2}\right)}^{1/2}\right]\geq 0$$

(23) $$g_{2}\left(x\right)=x_{2}^{2}x_{4}\left(0.2x_{1}+\frac{1}{12}x_{3}\right)-2.2\left\{8.94{\left[x_{1}+{\left(x_{3}^{2}-x_{2}^{2}\right)}^{1/2}\right]}^{3/4}\right\}\geq 0$$

(24) $$g_{3}\left(x\right)=x_{4}-0.0156x_{1}-0.15\geq 0$$

(25) $$g_{4}\left(x\right)=x_{4}-0.0156x_{3}-0.15\geq 0$$

(26) $$g_{5}\left(x\right)=x_{4}-1.05\geq 0$$

(27) $$g_{6}\left(x\right)=x_{4}-x_{2}\geq 0$$

where $x_{1}$, $x_{2}$, $x_{3}$, $x_{4}$ represent the width, depth, length, and thickness of the corrugated bulkhead plate, respectively; $g_{i}\left(x\right)$ represents the nonlinear inequality constraints.

**Figure 10:**
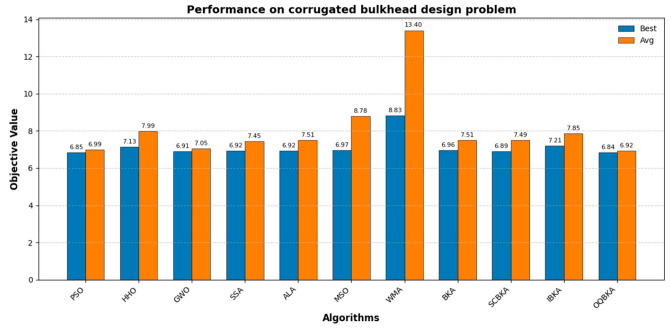
Performance on corrugated bulkhead design problem.

Table 7 and Figure 10 present the experimental results. From the results, it can be observed that OQBKA consistently achieved excellent optimal values and relatively low worst values across multiple independent runs, with a very small standard deviation, demonstrating good optimization accuracy, stability, and robustness.

(3) Reactor Network Design

**Figure 11:**
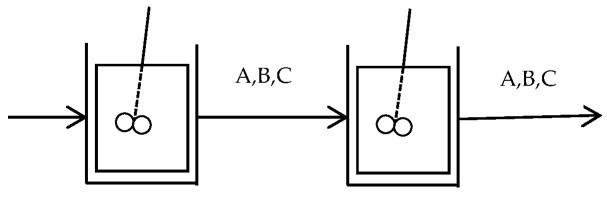
Schematic of a two-stage CSTR system in series. Letters above the arrows indicate stream composition: A (reactant), B (intermediate), C (final product).

The optimization of a two-stage continuous stirred-tank reactor (CSTR) system aims to maximize the concentration of substance B in the second reactor by adjusting the reactor parameters. As illustrated in Figure 11, species A is fed into the first reactor, where it is sequentially converted into intermediate B and final product C. The outlet streams from each reactor contain mixtures of species A, B, and C, as indicated by the labels on the connecting arrows. The mathematical model of the system can be formulated as follows:

Objective function:

(28) $$f(\bar{x})=x_{4}$$

The constraints are as follows:

(29) $$h_{1}(\bar{x})=k_{1}x_{5}x_{2}+x_{1}-1=0$$

(30) $$h_{2}(\bar{x})=k_{3}x_{5}x_{3}+x_{3}+x_{1}-1=0$$

(31) $$h_{3}(\bar{x})=k_{2}x_{6}x_{2}+x_{1}+x_{2}=0$$

(32) $$h_{4}(\bar{x})=k_{4}x_{6}x_{4}+x_{2}-x_{1}+x_{4}-x_{3}=0$$

(33) $$g_{1}(\bar{x})=x_{5}^{0.5}+x_{6}^{0.5}\leq 4$$

where $h_{i}(\bar{x})$ denotes nonlinear equality constraint functions and $g_{i}(\bar{x})$ denotes nonlinear inequality constraint functions. $x_{1}$ and $x_{2}$ represent the concentrations of substances A and B in the first vessel, respectively, with ranges $0\leq x_{1},x_{2}\leq 1$. $x_{3}$ and $x_{4}$ represent the concentrations of substances A and B in the second vessel, respectively, with ranges $0\leq x_{3},x_{4}\leq 1$. $x_{5}$ and $x_{6}$ denote the volumes of the first and second reactor vessels, respectively, with ranges $0.00001\leq x_{5},x_{6}\leq 16$. $k_{1}$ and $k_{2}$ denote the rate constants for the conversion of substance A to substance B in the first and second reactor vessels, respectively, with values $k_{1}=0.09755988$ and $k_{2}=0.99k_{1}$. $k_{3}$ and $k_{4}$ are additional rate constants in the first and second reactor vessels, respectively, with values $k_{3}=0.0397908$ and $k_{4}=0.9k_{3}$.

**Figure 12:**
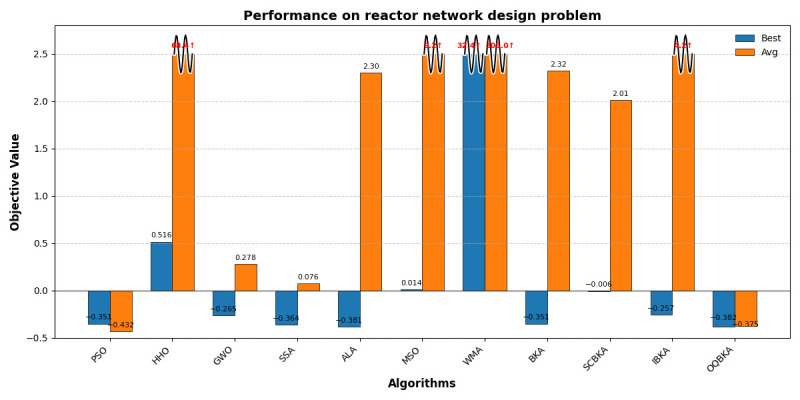
Performance on reactor network design problem. Red values with wavy lines and arrows indicate abnormally high objective values due to algorithmic divergence or poor performance.

As shown in Table 8, all listed values correspond to the objective function value of $-x_{4}$. Since the original objective function is $x_{4}$, maximizing $x_{4}$ is equivalent to minimizing $-x_{4}$. From the table, it can be observed that the OQBKA algorithm not only achieves the objective function value closest to the theoretical optimum but also outperforms other algorithms in terms of mean and standard deviation, demonstrating excellent stability. As shown in Figure 12, certain algorithms such as HHO, MSO, and WMA yield results that deviate significantly from the theoretical optimal range, suggesting that these methods may have failed to strictly satisfy all feasibility constraints during the optimization process, resulting in solutions that violate the mathematical model definitions. In contrast, the OQBKA algorithm consistently produces output values within a reasonable negative range throughout the optimization process, indicating stronger constraint-handling capability and higher solution reliability.

## 4. Conclusions

This study proposes an improved BKA, named OQBKA, which integrates opposition-based learning and a quasi-Newton strategy. By incorporating the convex lens opposition-based mechanism and the L-BFGS local refinement process, OQBKA effectively enhances the global exploration and local exploitation capabilities of the original BKA. Experimental results on the CEC2022 benchmark functions demonstrate that OQBKA achieves superior performance in both convergence accuracy and speed. Moreover, through engineering applications such as the step-cone pulley optimization, corrugated silo wall design, and reactor network design problems, OQBKA exhibits strong stability and high efficiency in solving various constrained optimization problems. These findings confirm its potential for addressing complex optimization tasks. Future research may further investigate the optimization and extended applications of OQBKA in higher-dimensional and more complex problem domains.

## Figures and Tables

**Table 1:** Comparative overview of BKA variants and their key design features.

Algorithm	Primary Enhancement Strategy	Role of OBL	Hybridization Framework	Core Characteristics
BWOA	WOA-based hybrid strategy with Lévy flight	None	Cross-algorithm hybrid	Avoids premature convergence and improves solution accuracy
OCBKA	OOA-based hybrid strategy with chaotic initialization	None	Cross-algorithm hybrid	Enhances global search capability
SCBKA	Sine–cosine guided search	None	Single-strategy enhancement	Improves convergence speed
IBKA	Gaussian mutation and oscillation with OBL	Escape from local optima	Multi-operator hybrid	Improves solution accuracy
BKAPI	PSO hybridization with DE mutation	None	Cross-algorithm hybrid	Avoids premature convergence
OQBKA	MOBL with L-BFGS refinement	Escape from local optima and maintain population diversity in the later stages of iteration	Multi-operator hybrid	Balances exploration and exploitation and improves solution accuracy

**Table 2:** Sensitivity analysis of the L-BFGS activation threshold k in OQBKA.

F(x)	*k*	Mean	Std
F1	0.1	3.00 × 10^2^	5.56 × 10^−9^
	0.4	3.00 × 10^2^	2.36 × 10^−9^
	0.7	**3.00 × 10^2^**	**2.24 × 10^−9^**
	0.9	3.00 × 10^2^	2.31 × 10^−9^
F5	0.1	9.71 × 10^2^	6.42 × 10^1^
	0.4	9.37 × 10^2^	1.96 × 10^1^
	0.7	**9.24 × 10^2^**	**1.56 × 10^1^**
	0.9	9.40 × 10^2^	7.83 × 10^1^
F8	0.1	2.23 × 10^3^	2.57 × 10^0^
	0.4	2.23 × 10^3^	4.19 × 10^0^
	0.7	**2.23 × 10^3^**	**2.32 × 10^0^**
	0.9	2.23 × 10^3^	2.34 × 10^0^
F11	0.1	2.62 × 10^3^	4.70 × 10^1^
	0.4	2.62 × 10^3^	5.85 × 10^1^
	0.7	**2.60 × 10^3^**	**1.74 × 10^0^**
	0.9	2.61 × 10^3^	2.74 × 10^1^

Bold values indicate the best performance in each column.

**Table 3:** Test results of different improved strategies.

F(x)		BKA	OBKA	LBKA	OQBKA
F1	Mean	7.94 × 10^8^	4.07 × 10^7^	**1.00 × 10^2^**	**1.00 × 10^2^**
	Std	2.42 × 10^8^	1.70 × 10^7^	**2.75 × 10^−7^**	1.97 × 10^−4^
	Rank	4	3	**1**	2
F2	Mean	1.20 × 10^3^	5.22 × 10^3^	**3.00 × 10^2^**	**3.00 × 10^2^**
	Std	1.24 × 10^3^	5.60 × 10^0^	1.05 × 10^−11^	**8.80 × 10^−12^**
	Rank	3	4	2	**1**
F3	Mean	4.97 × 10^2^	4.19 × 10^2^	**4.00 × 10^2^**	**4.00 × 10^2^**
	Std	1.28 × 10^2^	1.31 × 10^1^	**1.27 × 10^−13^**	3.94 × 10^−12^
	Rank	4	3	**1**	2
F4	Mean	**5.26 × 10^2^**	5.29 × 10^2^	5.51 × 10^2^	5.28 × 10^2^
	Std	5.58 × 10^0^	**1.82 × 10^0^**	2.88 × 10^1^	2.04 × 10^1^
	Rank	**1**	3	4	2
F5	Mean	6.33 × 10^2^	6.21 × 10^2^	6.38 × 10^2^	**6.06 × 10^2^**
	Std	**1.03 × 10^0^**	3.95 × 10^0^	2.82 × 10^0^	1.39 × 10^0^
	Rank	3	2	4	**1**
F6	Mean	7.65 × 10^2^	7.51 × 10^2^	7.80 × 10^2^	**7.39 × 10^2^**
	Std	**7.01 × 10^0^**	1.90 × 10^1^	7.37 × 10^0^	8.62 × 10^0^
	Rank	3	2	4	**1**
F7	Mean	8.24 × 10^2^	8.32 × 10^2^	8.31 × 10^2^	**8.21 × 10^2^**
	Std	6.84 × 10^−1^	8.39 × 10^0^	9.15 × 10^0^	**1.43 × 10^−5^**
	Rank	2	4	3	**1**
F8	Mean	1.13 × 10^3^	1.08 × 10^3^	1.30 × 10^3^	**9.35 × 10^2^**
	Std	**1.30 × 10^1^**	4.00 × 10^1^	6.92 × 10^1^	4.14 × 10^1^
	Rank	3	2	4	**1**
F9	Mean	1.83 × 10^3^	1.78 × 10^3^	1.98 × 10^3^	**1.42 × 10^3^**
	Std	3.26 × 10^2^	2.00 × 10^1^	**1.77 × 10^1^**	3.76 × 10^2^
	Rank	3	2	4	**1**
F10	Mean	1.15 × 10^3^	1.16 × 10^3^	1.19 × 10^3^	**1.11 × 10^3^**
	Std	1.37 × 10^1^	**3.16 × 10^0^**	8.37 × 10^1^	8.44 × 10^0^
	Rank	2	3	4	**1**
F11	Mean	1.24 × 10^6^	2.65 × 10^5^	**1.38 × 10^3^**	1.42 × 10^3^
	Std	1.72 × 10^6^	1.16 × 10^5^	**7.60 × 10^1^**	1.45 × 10^2^
	Rank	4	3	**1**	2
F12	Mean	4.10 × 10^3^	7.18 × 10^3^	**1.49 × 10^3^**	1.52 × 10^3^
	Std	2.68 × 10^3^	1.50 × 10^3^	1.51 × 10^2^	**1.70 × 10^1^**
	Rank	3	4	**1**	2
F13	Mean	1.48 × 10^3^	1.49 × 10^3^	1.46 × 10^3^	**1.45 × 10^3^**
	Std	4.27 × 10^1^	8.90 × 10^0^	2.78 × 10^1^	**6.81 × 10^0^**
	Rank	3	4	2	**1**
F14	Mean	4.58 × 10^3^	2.30 × 10^3^	1.62 × 10^3^	**1.55 × 10^3^**
	Std	4.23 × 10^3^	2.05 × 10^2^	1.57 × 10^2^	**1.63 × 10^1^**
	Rank	4	3	2	**1**
F15	Mean	1.80 × 10^3^	1.65 × 10^3^	1.67 × 10^3^	**1.60 × 10^3^**
	Std	7.11 × 10^1^	7.77 × 10^−1^	7.63 × 10^1^	**3.79 × 10^−3^**
	Rank	4	2	3	**1**
F16	Mean	1.77 × 10^3^	**1.75 × 10^3^**	1.76 × 10^3^	**1.75 × 10^3^**
	Std	2.09 × 10^1^	**2.66 × 10^0^**	2.99 × 10^1^	9.15 × 10^0^
	Rank	4	**1**	3	2
F17	Mean	3.12 × 10^3^	1.07 × 10^4^	1.94 × 10^3^	**1.91 × 10^3^**
	Std	1.40 × 10^3^	1.34 × 10^3^	7.13 × 10^1^	**4.93 × 10^1^**
	Rank	3	4	2	**1**
F18	Mean	2.00 × 10^3^	3.43 × 10^3^	1.96 × 10^3^	**1.95 × 10^3^**
	Std	5.06 × 10^1^	1.30 × 10^3^	4.03 × 10^1^	**5.28 × 10^0^**
	Rank	3	4	2	**1**
F19	Mean	2.06 × 10^3^	2.13 × 10^3^	2.17 × 10^3^	**2.04 × 10^3^**
	Std	5.27 × 10^1^	4.78 × 10^1^	1.11 × 10^2^	**1.50 × 10^1^**
	Rank	2	3	4	**1**
F20	Mean	2.32 × 10^3^	2.30 × 10^3^	**2.29 × 10^3^**	2.32 × 10^3^
	Std	**3.15 × 10^0^**	5.13 × 10^1^	1.17 × 10^2^	**3.15 × 10^0^**
	Rank	4	2	**1**	3
F21	Mean	2.36 × 10^3^	2.32 × 10^3^	**2.30 × 10^3^**	**2.30 × 10^3^**
	Std	6.40 × 10^1^	1.98 × 10^0^	**7.30 × 10^−1^**	1.23 × 10^0^
	Rank	4	3	2	**1**
F22	Mean	2.65 × 10^3^	2.63 × 10^3^	2.69 × 10^3^	**2.62 × 10^3^**
	Std	1.94 × 10^1^	**1.31 × 10^1^**	6.75 × 10^1^	1.50 × 10^1^
	Rank	3	2	4	**1**
F23	Mean	2.77 × 10^3^	2.75 × 10^3^	2.78 × 10^3^	**2.74 × 10^3^**
	Std	2.83 × 10^1^	7.00 × 10^0^	3.43 × 10^1^	**6.94 × 10^0^**
	Rank	3	2	4	**1**
F24	Mean	**2.91 × 10^3^**	2.94 × 10^3^	2.92 × 10^3^	2.92 × 10^3^
	Std	**1.32 × 10^0^**	9.34 × 10^0^	3.65 × 10^1^	3.36 × 10^1^
	Rank	**1**	4	3	2
F25	Mean	3.08 × 10^3^	3.12 × 10^3^	3.03 × 10^3^	**2.90 × 10^3^**
	Std	1.95 × 10^2^	2.37 × 10^0^	3.80 × 10^1^	**8.49 × 10^−8^**
	Rank	3	4	2	**1**
F26	Mean	3.12 × 10^3^	3.09 × 10^3^	3.10 × 10^3^	**3.08 × 10^3^**
	Std	3.84 × 10^0^	**7.68 × 10^−1^**	2.02 × 10^1^	4.99 × 10^0^
	Rank	4	2	3	**1**
F27	Mean	3.41 × 10^3^	3.27 × 10^3^	**3.14 × 10^3^**	**3.14 × 10^3^**
	Std	**3.60 × 10^0^**	3.76 × 10^1^	5.99 × 10^1^	5.99 × 10^1^
	Rank	4	3	**1**	2
F28	Mean	3.25 × 10^3^	3.21 × 10^3^	3.29 × 10^3^	**3.19 × 10^3^**
	Std	1.13 × 10^2^	6.32 × 10^1^	**3.36 × 10^1^**	6.89 × 10^1^
	Rank	3	2	4	**1**
F29	Mean	4.89 × 10^4^	4.87 × 10^5^	3.63 × 10^3^	**3.44 × 10^3^**
	Std	6.41 × 10^4^	1.26 × 10^5^	8.22 × 10^1^	**1.20 × 10^1^**
	Rank	3	4	2	**1**
Average Rank		3.10	2.90	2.66	**1.34**

Bold values indicate the best performance in each row.

**Table 4:** Comparison of test results on CEC2022 test functions (dim = 10).

F(x)		PSO	HHO	GWO	SSA	ALA	MSO	WMA	BKA	SCBKA	IBKA	OQBKA
F1	Mean	5.95 × 10^2^	5.07 × 10^3^	5.13 × 10^3^	2.18 × 10^3^	9.30 × 10^2^	1.09 × 10^4^	3.39 × 10^2^	1.40 × 10^3^	9.99 × 10^2^	6.85 × 10^3^	**3.00 × 10^2^**
	Rank	3	8	9	7	4	11	2	6	5	10	**1**
	Std	2.91 × 10^2^	9.88 × 10^2^	3.43 × 10^3^	1.69 × 10^3^	6.43 × 10^2^	6.14 × 10^3^	1.93 × 10^2^	2.40 × 10^3^	1.41 × 10^3^	3.07 × 10^3^	**1.48 × 10^−11^**
	Wilcoxon	3.02 × 10^−11^	3.02 × 10^−11^	3.02 × 10^−11^	3.02 × 10^−11^	3.02 × 10^−11^	3.02 × 10^−11^	3.02 × 10^−11^	3.02 × 10^−11^	3.02 × 10^−11^	3.02 × 10^−11^	N/A
	+/−/=	−	−	−	−	−	−	−	−	−	−	N/A
F2	Mean	4.22 × 10^2^	5.05 × 10^2^	4.38 × 10^2^	4.16 × 10^2^	4.10 × 10^2^	4.35 × 10^2^	4.09 × 10^2^	4.17 × 10^2^	4.26 × 10^2^	4.13 × 10^2^	**4.00 × 10^2^**
	Rank	7	11	10	5	3	9	2	6	8	4	**1**
	Std	2.47 × 10^1^	9.88 × 10^1^	2.62 × 10^1^	2.44 × 10^1^	1.55 × 10^1^	3.14 × 10^1^	1.24 × 10^1^	2.59 × 10^1^	3.90 × 10^1^	1.33 × 10^1^	**1.04 × 10^−11^**
	Wilcoxon	1.33 × 10^−10^	4.97 × 10^−11^	3.02 × 10^−11^	3.47 × 10^−10^	4.97 × 10^−11^	4.97 × 10^−11^	3.02 × 10^−11^	1.41 × 10^−9^	8.15 × 10^−11^	4.97 × 10^−11^	N/A
	+/−/=	−	−	−	−	−	−	−	−	−	−	N/A
F3	Mean	**6.00 × 10^2^**	6.42 × 10^2^	6.03 × 10^2^	6.05 × 10^2^	6.01 × 10^2^	6.19 × 10^2^	6.01 × 10^2^	6.28 × 10^2^	6.28 × 10^2^	6.06 × 10^2^	6.07 × 10^2^
	Rank	**1**	11	4	5	3	8	2	10	9	6	7
	Std	**1.12 × 10^0^**	1.23 × 10^1^	2.81 × 10^0^	7.37 × 10^0^	1.23 × 10^0^	8.93 × 10^0^	1.30 × 10^0^	9.07 × 10^0^	9.78 × 10^0^	1.95 × 10^0^	2.50 × 10^0^
	Wilcoxon	6.72 × 10^−10^	3.02 × 10^−11^	1.00 × 10^−4^	3.68 × 10^−2^	8.10 × 10^−10^	1.41 × 10^−9^	3.02 × 10^−11^	3.02 × 10^−11^	3.02 × 10^−11^	1.06 × 10^−1^	N/A
	+/−/=	+	−	+	+	+	−	+	−	−	=	N/A
F4	Mean	**8.15 × 10^2^**	8.28 × 10^2^	8.20 × 10^2^	8.29 × 10^2^	8.22 × 10^2^	8.27 × 10^2^	8.26 × 10^2^	8.20 × 10^2^	8.24 × 10^2^	8.25 × 10^2^	8.23 × 10^2^
	Rank	**1**	10	2	11	4	9	8	3	6	7	5
	Std	7.61 × 10^0^	7.44 × 10^0^	1.04 × 10^1^	9.54 × 10^0^	6.74 × 10^0^	1.03 × 10^1^	9.72 × 10^0^	8.52 × 10^0^	8.34 × 10^0^	9.85 × 10^0^	**5.40 × 10^0^**
	Wilcoxon	6.00 × 10^−4^	6.00 × 10^−3^	6.87 × 10^−2^	1.75 × 10^−2^	5.17 × 10^−1^	8.59 × 10^−2^	1.71 × 10^−1^	2.85 × 10^−2^	6.58 × 10^−1^	7.50 × 10^−1^	N/A
	+/−/=	+	−	=	−	=	=	=	+	=	=	N/A
F5	Mean	**9.01 × 10^2^**	1.47 × 10^3^	9.33 × 10^2^	1.29 × 10^3^	9.20 × 10^2^	1.21 × 10^3^	9.07 × 10^2^	1.08 × 10^3^	1.10 × 10^3^	9.67 × 10^2^	9.62 × 10^2^
	Rank	**1**	11	4	10	3	9	2	7	8	6	5
	Std	**1.30 × 10^0^**	1.89 × 10^2^	6.00 × 10^1^	2.33 × 10^2^	2.38 × 10^1^	2.40 × 10^2^	1.01 × 10^1^	8.82 × 10^1^	1.10 × 10^2^	5.05 × 10^1^	7.16 × 10^1^
	Wilcoxon	2.61 × 10^−10^	3.16 × 10^−10^	6.00 × 10^−3^	1.56 × 10^−8^	1.40 × 10^−3^	5.00 × 10^−9^	1.78 × 10^−10^	1.01 × 10^−8^	1.10 × 10^−8^	3.29 × 10^−1^	N/A
	+/−/=	+	−	+	−	+	−	+	−	−	=	N/A
F6	Mean	4.31 × 10^3^	1.61 × 10^4^	1.16 × 10^4^	4.53 × 10^3^	3.94 × 10^3^	3.78 × 10^3^	4.62 × 10^3^	3.59 × 10^3^	4.45 × 10^3^	6.34 × 10^4^	**1.83 × 10^3^**
	Rank	5	10	9	7	4	3	8	2	6	11	**1**
	Std	2.48 × 10^3^	1.58 × 10^4^	6.20 × 10^3^	2.21 × 10^3^	2.06 × 10^3^	1.93 × 10^3^	2.11 × 10^3^	2.10 × 10^3^	2.03 × 10^3^	1.65 × 10^5^	**2.22 × 10^1^**
	Wilcoxon	4.08 × 10^−11^	3.02 × 10^−11^	3.02 × 10^−11^	9.76 × 10^−10^	3.02 × 10^−11^	9.76 × 10^−10^	3.02 × 10^−11^	3.69 × 10^−11^	7.39 × 10^−11^	3.02 × 10^−11^	N/A
	+/−/=	−	−	−	−	−	−	−	−	−	−	N/A
F7	Mean	**2.02 × 10^3^**	2.09 × 10^3^	2.04 × 10^3^	2.03 × 10^3^	2.03 × 10^3^	2.06 × 10^3^	**2.02 × 10^3^**	2.06 × 10^3^	2.05 × 10^3^	2.03 × 10^3^	2.03 × 10^3^
	Rank	**1**	11	7	6	3	10	2	9	8	5	4
	Std	7.89 × 10^0^	2.96 × 10^1^	1.31 × 10^1^	2.65 × 10^1^	7.67 × 10^0^	3.92 × 10^1^	8.69 × 10^0^	2.36 × 10^1^	2.23 × 10^1^	**7.14 × 10^0^**	8.59 × 10^0^
	Wilcoxon	5.09 × 10^−6^	1.33 × 10^−10^	1.25 × 10^−2^	8.45 × 10^−1^	1.92 × 10^−1^	1.00 × 10^−4^	7.30 × 10^−3^	4.20 × 10^−10^	8.84 × 10^−7^	1.16 × 10^−1^	N/A
	+/−/=	+	−	−	=	=	−	+	−	−	=	N/A
F8	Mean	2.24 × 10^3^	2.24 × 10^3^	2.23 × 10^3^	2.23 × 10^3^	**2.22 × 10^3^**	2.25 × 10^3^	2.24 × 10^3^	2.23 × 10^3^	**2.22 × 10^3^**	2.23 × 10^3^	**2.22 × 10^3^**
	Rank	9	10	6	7	3	11	8	5	2	4	**1**
	Std	3.78 × 10^1^	1.34 × 10^1^	2.20 × 10^1^	3.78 × 10^1^	3.71 × 10^0^	4.86 × 10^1^	2.54 × 10^1^	2.29 × 10^1^	5.32 × 10^0^	4.96 × 10^0^	**3.19 × 10^0^**
	Wilcoxon	8.97 × 10^−2^	3.37 × 10^−5^	1.78 × 10^−4^	4.53 × 10^−1^	6.87 × 10^−2^	6.56 × 10^−2^	3.02 × 10^−11^	1.00 × 10^−3^	4.95 × 10^−2^	4.40 × 10^−3^	N/A
	+/−/=	=	−	−	=	=	=	−	−	−	−	N/A
F9	Mean	2.55 × 10^3^	2.66 × 10^3^	2.59 × 10^3^	2.54 × 10^3^	2.53 × 10^3^	2.56 × 10^3^	2.53 × 10^3^	2.57 × 10^3^	2.55 × 10^3^	2.53 × 10^3^	**2.49 × 10^3^**
	Rank	6	11	10	5	3	8	2	9	7	4	**1**
	Std	4.68 × 10^1^	4.44 × 10^1^	3.78 × 10^1^	3.73 × 10^1^	1.70 × 10^−1^	1.86 × 10^1^	**8.44 × 10^−14^**	4.31 × 10^1^	3.09 × 10^1^	2.56 × 10^0^	1.04 × 10^−10^
	Wilcoxon	2.97 × 10^−11^	3.02 × 10^−11^	3.02 × 10^−11^	1.71 × 10^−11^	3.02 × 10^−11^	3.02 × 10^−11^	3.02 × 10^−11^	3.02 × 10^−11^	3.02 × 10^−11^	3.02 × 10^−11^	N/A
	+/−/=	−	−	−	−	−	−	−	−	−	−	N/A
F10	Mean	2.57 × 10^3^	2.64 × 10^3^	2.56 × 10^3^	2.64 × 10^3^	2.58 × 10^3^	2.58 × 10^3^	2.58 × 10^3^	2.61 × 10^3^	2.57 × 10^3^	**2.51 × 10^3^**	**2.51 × 10^3^**
	Rank	4	10	3	11	6	7	8	9	5	**1**	2
	Std	6.64 × 10^1^	2.40 × 10^2^	6.11 × 10^1^	2.09 × 10^2^	1.80 × 10^2^	1.15 × 10^2^	7.86 × 10^1^	1.58 × 10^2^	6.96 × 10^1^	**3.18 × 10^1^**	3.51 × 10^1^
	Wilcoxon	1.00 × 10^−4^	4.20 × 10^−10^	1.32 × 10^−2^	6.28 × 10^−6^	2.07 × 10^−2^	3.20 × 10^−9^	4.00 × 10^−4^	2.78 × 10^−7^	8.84 × 10^−7^	8.59 × 10^−2^	N/A
	+/−/=	−	−	−	−	−	−	−	−	−	=	N/A
F11	Mean	2.86 × 10^3^	2.99 × 10^3^	3.04 × 10^3^	2.81 × 10^3^	2.83 × 10^3^	3.00 × 10^3^	2.93 × 10^3^	2.81 × 10^3^	2.79 × 10^3^	2.96 × 10^3^	**2.60 × 10^3^**
	Rank	6	9	11	3	5	10	7	4	2	8	**1**
	Std	1.86 × 10^2^	2.47 × 10^2^	1.51 × 10^2^	1.34 × 10^2^	1.06 × 10^2^	3.95 × 10^2^	2.74 × 10^2^	1.56 × 10^2^	1.45 × 10^2^	1.32 × 10^2^	**7.63 × 10^−7^**
	Wilcoxon	2.87 × 10^−10^	3.69 × 10^−11^	3.69 × 10^−11^	8.15 × 10^−11^	9.92 × 10^−11^	3.02 × 10^−11^	3.02 × 10^−11^	1.46 × 10^−10^	8.10 × 10^−10^	4.50 × 10^−11^	N/A
	+/−/=	−	−	−	−	−	−	−	−	−	−	N/A
F12	Mean	2.87 × 10^3^	2.95 × 10^3^	2.87 × 10^3^	2.88 × 10^3^	2.86 × 10^3^	2.88 × 10^3^	2.87 × 10^3^	2.87 × 10^3^	2.87 × 10^3^	2.86 × 10^3^	**2.85 × 10^3^**
	Rank	8	11	6	9	2	10	4	7	5	3	**1**
	Std	1.30 × 10^1^	5.95 × 10^1^	1.00 × 10^1^	4.05 × 10^1^	1.79 × 10^0^	1.62 × 10^1^	1.89 × 10^0^	1.30 × 10^1^	7.41 × 10^0^	**9.30 × 10^−1^**	1.09 × 10^0^
	Wilcoxon	2.99 × 10^−11^	3.02 × 10^−11^	3.02 × 10^−11^	3.01 × 10^−11^	3.02 × 10^−11^	3.02 × 10^−11^	3.02 × 10^−11^	3.02 × 10^−11^	3.02 × 10^−11^	3.02 × 10^−11^	N/A
	+/−/=	−	−	−	−	−	−	−	−	−	−	N/A
Average Rank		4.33	10.3	6.75	7.17	3.58	8.75	4.58	6.42	5.92	5.75	**2.50**

Bold values indicate the best performance in each row.

**Table 5:** Comparison of test results on CEC2022 test functions (dim = 20).

F(x)		PSO	HHO	GWO	SSA	ALA	MSO	WMA	BKA	SCBKA	IBKA	OQBKA
F1	Mean	2.38 × 10^4^	4.78 × 10^4^	2.22 × 10^4^	5.05 × 10^4^	1.99 × 10^4^	4.20 × 10^4^	1.98 × 10^4^	1.43 × 10^4^	1.51 × 10^4^	5.02 × 10^4^	**3.00 × 10^2^**
	Rank	7	9	6	11	5	8	4	2	3	10	**1**
	Std	1.06 × 10^4^	1.74 × 10^4^	6.38 × 10^3^	1.67 × 10^4^	6.09 × 10^3^	1.27 × 10^4^	8.25 × 10^3^	7.44 × 10^3^	1.08 × 10^4^	1.19 × 10^4^	**1.79 × 10^−9^**
	Wilcoxon	3.02 × 10^−11^	3.02 × 10^−11^	3.02 × 10^−11^	3.02 × 10^−11^	3.02 × 10^−11^	3.02 × 10^−11^	3.02 × 10^−11^	3.02 × 10^−11^	3.02 × 10^−11^	3.02 × 10^−11^	N/A
	+/−/=	−	−	−	−	−	−	−	−	−	−	N/A
F2	Mean	4.86 × 10^2^	7.26 × 10^2^	5.17 × 10^2^	4.64 × 10^2^	4.76 × 10^2^	5.46 × 10^2^	4.58 × 10^2^	8.24 × 10^2^	7.48 × 10^2^	5.73 × 10^2^	**4.00 × 10^2^**
	Rank	5	9	6	3	4	7	2	11	10	8	**1**
	Std	5.40 × 10^1^	8.88 × 10^1^	5.08 × 10^1^	2.23 × 10^1^	2.86 × 10^1^	6.29 × 10^1^	1.75 × 10^1^	4.09 × 10^2^	3.73 × 10^2^	6.17 × 10^1^	**7.01 × 10^−11^**
	Wilcoxon	3.02 × 10^−11^	3.02 × 10^−11^	3.02 × 10^−11^	3.02 × 10^−11^	3.02 × 10^−11^	3.02 × 10^−11^	3.02 × 10^−11^	3.02 × 10^−11^	3.02 × 10^−11^	3.02 × 10^−11^	N/A
	+/−/=	−	−	−	−	−	−	−	−	−	−	N/A
F3	Mean	**6.06 × 10^2^**	6.65 × 10^2^	6.10 × 10^2^	6.33 × 10^2^	6.14 × 10^2^	6.38 × 10^2^	6.12 × 10^2^	6.58 × 10^2^	6.52 × 10^2^	6.27 × 10^2^	6.34 × 10^2^
	Rank	**1**	11	2	6	4	8	3	10	9	5	7
	Std	**4.25 × 10^0^**	9.20 × 10^0^	4.55 × 10^0^	1.34 × 10^1^	6.22 × 10^0^	1.11 × 10^1^	6.70 × 10^0^	1.30 × 10^1^	9.83 × 10^0^	4.39 × 10^0^	1.33 × 10^1^
	Wilcoxon	4.40 × 10^−4^	1.83 × 10^−4^	1.01 × 10^−3^	7.97 × 10^−1^	6.74 × 10^−6^	2.29 × 10^−1^	1.83 × 10^−4^	1.83 × 10^−4^	1.83 × 10^−4^	5.19 × 10^−2^	N/A
	+/−/=	+	−	+	=	+	=	+	−	−	=	N/A
F4	Mean	**8.66 × 10^2^**	8.91 × 10^2^	8.68 × 10^2^	8.90 × 10^2^	8.84 × 10^2^	8.79 × 10^2^	9.06 × 10^2^	8.87 × 10^2^	8.83 × 10^2^	9.03 × 10^2^	8.83 × 10^2^
	Rank	**1**	9	2	8	6	3	11	7	5	10	4
	Std	2.82 × 10^1^	1.06 × 10^1^	3.00 × 10^1^	1.92 × 10^1^	2.42 × 10^1^	2.33 × 10^1^	3.33 × 10^1^	1.82 × 10^1^	1.77 × 10^1^	2.13 × 10^1^	**3.79 × 10^0^**
	Wilcoxon	1.60 × 10^−3^	3.40 × 10^−3^	4.07 × 10^−2^	2.70 × 10^−2^	9.75 × 10^−1^	3.82 × 10^−1^	3.00 × 10^−3^	3.09 × 10^−1^	8.45 × 10^−1^	5.57 × 10^−10^	N/A
	+/−/=	+	−	+	−	=	=	−	=	=	−	N/A
F5	Mean	**1.06 × 10^3^**	3.22 × 10^3^	1.35 × 10^3^	2.43 × 10^3^	1.91 × 10^3^	2.36 × 10^3^	1.24 × 10^3^	2.38 × 10^3^	2.46 × 10^3^	2.74 × 10^3^	2.20 × 10^3^
	Rank	**1**	11	3	8	4	6	2	7	9	10	5
	Std	3.46 × 10^2^	3.52 × 10^2^	3.52 × 10^2^	1.77 × 10^2^	6.19 × 10^2^	6.38 × 10^2^	3.92 × 10^2^	4.41 × 10^2^	5.37 × 10^2^	7.28 × 10^2^	**1.59 × 10^2^**
	Wilcoxon	5.80 × 10^−3^	3.02 × 10^−11^	3.08 × 10^−8^	2.44 × 10^−9^	2.30 × 10^−2^	3.93 × 10^−1^	3.02 × 10^−11^	2.70 × 10^−2^	1.50 × 10^−3^	8.00 × 10^−4^	N/A
	+/−/=	+	−	+	−	+	=	+	−	−	−	N/A
F6	Mean	1.50 × 10^6^	1.51 × 10^6^	7.92 × 10^6^	6.51 × 10^3^	2.97 × 10^4^	7.26 × 10^3^	1.10 × 10^4^	3.00 × 10^7^	6.75 × 10^6^	3.80 × 10^6^	**1.86 × 10^3^**
	Rank	6	7	10	2	5	3	4	11	9	8	**1**
	Std	7.88 × 10^6^	1.87 × 10^6^	1.51 × 10^7^	4.48 × 10^3^	4.12 × 10^4^	5.58 × 10^3^	7.56 × 10^3^	1.18 × 10^8^	2.29 × 10^7^	7.49 × 10^6^	**1.26 × 10^1^**
	Wilcoxon	3.02 × 10^−11^	3.02 × 10^−11^	3.02 × 10^−11^	6.07 × 10^−11^	3.02 × 10^−11^	7.39 × 10^−11^	3.02 × 10^−11^	3.02 × 10^−11^	3.02 × 10^−11^	3.02 × 10^−11^	N/A
	+/−/=	−	−	−	−	−	−	−	−	−	−	N/A
F7	Mean	**2.09 × 10^3^**	2.22 × 10^3^	2.12 × 10^3^	2.14 × 10^3^	2.12 × 10^3^	2.14 × 10^3^	2.11 × 10^3^	2.14 × 10^3^	2.14 × 10^3^	2.13 × 10^3^	**2.09 × 10^3^**
	Rank	**1**	11	4	7	5	10	3	8	9	6	2
	Std	5.00 × 10^1^	7.35 × 10^1^	4.94 × 10^1^	6.46 × 10^1^	4.28 × 10^1^	7.36 × 10^1^	4.98 × 10^1^	4.32 × 10^1^	4.07 × 10^1^	2.75 × 10^1^	**2.06 × 10^1^**
	Wilcoxon	2.99 × 10^−1^	3.47 × 10^−10^	3.50 × 10^−2^	2.40 × 10^−3^	1.75 × 10^−2^	2.60 × 10^−3^	3.49 × 10^−1^	6.00 × 10^−4^	2.53 × 10^−4^	2.60 × 10^−5^	N/A
	+/−/=	=	−	−	−	−	−	=	−	−	−	N/A
F8	Mean	2.29 × 10^3^	2.33 × 10^3^	2.29 × 10^3^	2.29 × 10^3^	2.25 × 10^3^	2.32 × 10^3^	2.31 × 10^3^	2.29 × 10^3^	2.25 × 10^3^	2.24 × 10^3^	**2.23 × 10^3^**
	Rank	6	11	8	5	3	10	9	7	4	2	**1**
	Std	6.65 × 10^1^	1.07 × 10^2^	7.41 × 10^1^	5.89 × 10^1^	2.56 × 10^1^	1.07 × 10^2^	6.41 × 10^1^	7.55 × 10^1^	3.22 × 10^1^	9.02 × 10^0^	**5.02 × 10^0^**
	Wilcoxon	4.00 × 10^−4^	9.21 × 10^−5^	1.50 × 10^−3^	3.00 × 10^−4^	1.86 × 10^−1^	2.00 × 10^−4^	3.02 × 10^−11^	1.44 × 10^−3^	4.00 × 10^−4^	3.76 × 10^−2^	N/A
	+/−/=	−	−	−	−	−	−	−	−	−	−	N/A
F9	Mean	2.51 × 10^3^	2.65 × 10^3^	2.53 × 10^3^	2.48 × 10^3^	2.49 × 10^3^	2.49 × 10^3^	2.48 × 10^3^	2.60 × 10^3^	2.57 × 10^3^	2.50 × 10^3^	**2.47 × 10^3^**
	Rank	7	11	8	3	4	5	2	10	9	6	**1**
	Std	4.94 × 10^1^	7.07 × 10^1^	4.29 × 10^1^	8.14 × 10^−2^	6.64 × 10^0^	6.59 × 10^0^	2.11 × 10^−2^	7.05 × 10^1^	6.05 × 10^1^	7.62 × 10^0^	**4.22 × 10^−10^**
	Wilcoxon	3.02 × 10^−11^	3.02 × 10^−11^	3.02 × 10^−11^	3.02 × 10^−11^	3.02 × 10^−11^	3.02 × 10^−11^	3.02 × 10^−11^	3.02 × 10^−11^	3.02 × 10^−11^	3.02 × 10^−11^	N/A
	+/−/=	−	−	−	−	−	−	−	−	−	−	N/A
F10	Mean	3.25 × 10^3^	4.72 × 10^3^	3.64 × 10^3^	3.89 × 10^3^	4.21 × 10^3^	3.54 × 10^3^	4.24 × 10^3^	4.31 × 10^3^	4.07 × 10^3^	3.48 × 10^3^	**2.86 × 10^3^**
	Rank	2	11	5	6	8	4	9	10	7	3	**1**
	Std	**5.22 × 10^2^**	1.10 × 10^3^	1.07 × 10^3^	7.15 × 10^2^	1.01 × 10^3^	5.64 × 10^2^	1.64 × 10^3^	1.05 × 10^3^	8.45 × 10^2^	1.15 × 10^3^	7.17 × 10^2^
	Wilcoxon	1.40 × 10^−2^	1.21 × 10^−10^	2.40 × 10^−3^	1.00 × 10^−4^	1.00 × 10^−4^	1.80 × 10^−3^	2.10 × 10^−3^	1.86 × 10^−9^	1.43 × 10^−8^	4.00 × 10^−4^	N/A
	+/−/=	−	−	−	−	−	−	−	−	−	−	N/A
F11	Mean	3.01 × 10^3^	5.27 × 10^3^	3.79 × 10^3^	2.94 × 10^3^	3.89 × 10^3^	4.30 × 10^3^	2.90 × 10^3^	5.50 × 10^3^	5.36 × 10^3^	6.32 × 10^3^	**2.80 × 10^3^**
	Rank	4	8	5	3	6	7	2	10	9	11	**1**
	Std	3.25 × 10^1^	8.12 × 10^2^	4.40 × 10^2^	1.26 × 10^2^	4.20 × 10^2^	5.37 × 10^2^	**5.41 × 10^0^**	1.24 × 10^3^	1.26 × 10^3^	8.19 × 10^2^	1.47 × 10^2^
	Wilcoxon	2.38 × 10^−7^	3.02 × 10^−11^	3.02 × 10^−11^	1.17 × 10^−3^	3.02 × 10^−11^	3.02 × 10^−11^	3.02 × 10^−11^	3.02 × 10^−11^	3.02 × 10^−11^	3.02 × 10^−11^	N/A
	+/−/=	−	−	−	−	−	−	−	−	−	−	N/A
F12	Mean	3.02 × 10^3^	3.33 × 10^3^	2.99 × 10^3^	2.99 × 10^3^	2.97 × 10^3^	3.03 × 10^3^	2.99 × 10^3^	3.12 × 10^3^	3.06 × 10^3^	2.97 × 10^3^	**2.90 × 10^3^**
	Rank	7	11	6	5	2	8	4	10	9	3	**1**
	Std	7.81 × 10^1^	1.91 × 10^2^	2.89 × 10^1^	3.32 × 10^1^	3.07 × 10^1^	5.82 × 10^1^	3.77 × 10^1^	9.62 × 10^1^	7.63 × 10^1^	1.06 × 10^1^	**3.96 × 10^0^**
	Wilcoxon	4.08 × 10^−11^	3.02 × 10^−11^	4.08 × 10^−11^	6.07 × 10^−11^	5.57 × 10^−10^	3.34 × 10^−11^	3.02 × 10^−11^	3.34 × 10^−11^	3.34 × 10^−11^	5.49 × 10^−11^	N/A
	+/−/=	−	−	−	−	−	−	−	−	−	−	N/A
Average Rank		4.00	9.92	5.42	5.58	4.67	6.58	4.58	8.58	7.67	6.83	**2.17**

Bold values indicate the best performance in each row.

**Table 6:** Experimental results of the step-cone pulley problem.

	PSO	HHO	GWO	SSA	ALA	MSO	WMA	BKA	SCBKA	IBKA	OQBKA
Best	1.63 × 10^1^	1.84 × 10^1^	1.63 × 10^1^	1.63 × 10^1^	1.63 × 10^1^	1.67 × 10^1^	2.73 × 10^1^	1.63 × 10^1^	1.66 × 10^1^	1.65 × 10^1^	**1.62 × 10^1^**
Worst	2.19 × 10^1^	2.12 × 10^2^	1.69 × 10^1^	1.74 × 10^1^	1.84 × 10^1^	2.38 × 10^1^	4.73 × 10^7^	3.36 × 10^1^	5.63 × 10^1^	1.85 × 10^1^	**1.66 × 10^1^**
Std	1.69 × 10^0^	6.83 × 10^1^	1.82 × 10^−1^	2.86 × 10^−1^	4.77 × 10^−1^	1.70 × 10^0^	1.45 × 10^7^	4.15 × 10^0^	9.28 × 10^0^	5.62 × 10^−1^	**8.70 × 10^−2^**
Avg	1.74 × 10^1^	5.69 × 10^1^	1.66 × 10^1^	1.65 × 10^1^	1.68 × 10^1^	1.86 × 10^1^	6.33 × 10^6^	1.96 × 10^1^	2.17 × 10^1^	1.73 × 10^1^	**1.63 × 10^1^**

Bold values indicate the best performance in each row.

**Table 7:** Experimental results of the corrugated bulkhead design problem.

	PSO	HHO	GWO	SSA	ALA	MSO	WMA	BKA	SCBKA	IBKA	OQBKA
Best	6.85 × 10^0^	7.13 × 10^0^	6.91 × 10^0^	6.92 × 10^0^	6.92 × 10^0^	6.97 × 10^0^	8.83 × 10^0^	6.96 × 10^0^	6.89 × 10^0^	7.21 × 10^0^	**6.84 × 10^0^**
Worst	7.41 × 10^0^	9.85 × 10^0^	7.29 × 10^0^	1.03 × 10^1^	9.13 × 10^0^	1.27 × 10^1^	1.94 × 10^1^	9.06 × 10^0^	9.10 × 10^0^	9.29 × 10^0^	**7.17 × 10^0^**
Std	1.34 × 10^−1^	7.37 × 10^−1^	1.09 × 10^−1^	7.51 × 10^−1^	5.47 × 10^−1^	1.35 × 10^0^	3.33 × 10^0^	5.37 × 10^−1^	5.32 × 10^−1^	5.80 × 10^−1^	**9.02 × 10^−2^**
Avg	6.99 × 10^0^	7.99 × 10^0^	7.05 × 10^0^	7.45 × 10^0^	7.51 × 10^0^	8.78 × 10^0^	1.34 × 10^1^	7.51 × 10^0^	7.49 × 10^0^	7.85 × 10^0^	**6.92 × 10^0^**

Bold values indicate the best performance in each row.

**Table 8:** Experimental results of the reactor network design problem.

	PSO	HHO	GWO	SSA	ALA	MSO	WMA	BKA	SCBKA	IBKA	OQBKA
Best	−3.51 × 10^−1^	5.16 × 10^−1^	−2.65 × 10^−1^	−3.64 × 10^−1^	−3.81 × 10^−1^	1.37 × 10^−2^	3.24 × 10^1^	−3.51 × 10^−1^	−5.56 × 10^−3^	−2.57 × 10^−1^	**−3.82 × 10^−1^**
Worst	3.24 × 10^−1^	1.54 × 10^2^	8.74 × 10^−1^	2.34 × 10^0^	3.71 × 10^1^	4.45 × 10^1^	1.01 × 10^3^	3.29 × 10^1^	1.33 × 10^1^	1.33 × 10^1^	**−3.74 × 10^−1^**
Std	2.07 × 10^−1^	4.67 × 10^1^	3.60 × 10^−1^	6.31 × 10^−1^	8.26 × 10^0^	1.22 × 10^1^	2.42 × 10^2^	7.40 × 10^0^	3.74 × 10^0^	4.81 × 10^0^	**2.41 × 10^−3^**
Avg	−4.32 × 10^−2^	6.84 × 10^1^	2.78 × 10^−1^	7.56 × 10^−2^	2.30 × 10^0^	9.15 × 10^0^	3.05 × 10^2^	2.32 × 10^0^	2.01 × 10^0^	4.24 × 10^0^	−**3.75 × 10^−1^**

Bold values indicate the best performance in each row.

## Data Availability

The data that support the findings of this study are available from the corresponding author upon request. There are no restrictions on data availability.
